# CDP and RDP as Alternatives to TPhP: Comparative Effects on Glucose Homeostasis and Hepatic and Whole-Pancreas Molecular Endpoints in Mice

**DOI:** 10.3390/toxics14100841

**Published:** 2026-09-22

**Authors:** Jiajun Chang, Zhuozhuo Wang, Yao Xiao, Yifan Liu, Yuan Meng, Qinghui Huang, Zhiliang Zhu, Daqiang Yin, Xiaojuan Xu, Yanling Qiu

**Affiliations:** 1 Key Laboratory of Yangtze River Water Environment, College of Environmental Science and Engineering, Tongji University, 1239 Siping Road, Shanghai 200092, China; 2210091@tongji.edu.cn (J.C.); 2210526@tongji.edu.cn (Z.W.); 2111065@tongji.edu.cn (Y.X.); qhhuang@tongji.edu.cn (Q.H.); zzl@tongji.edu.cn (Z.Z.); yindq@tongji.edu.cn (D.Y.); 2 Shanghai Institute of Pollution Control and Ecological Security, College of Environmental Science and Engineering, Tongji University, 1239 Siping Road, Shanghai 200092, China; 3 Department of Pathology and Pathophysiology, Teaching Laboratory Center, School of Medicine, Tongji University, Shanghai 200331, China; liuyifan170909@163.com; 4 Department of Criminal Science and Technology, Shandong Police College, Jinan 250200, China; yuanmeng0419@163.com

**Keywords:** aryl organophosphate flame retardants, triphenyl phosphate, cresyl diphenyl phosphate, resorcinol bis(diphenyl phosphate), glucose homeostasis, hepatic insulin signaling, whole-pancreas transcriptomics

## Abstract

Organophosphate flame retardants (OPFRs) are widely used as replacements for brominated flame retardants, yet the metabolic effects of cresyl diphenyl phosphate (CDP) and resorcinol bis(diphenyl phosphate) (RDP), two structurally related alternatives to the major aryl OPFR triphenyl phosphate (TPhP), remain poorly characterized. Male C57BL/6J mice received TPhP, CDP, or RDP by oral gavage every 3 days for 70 days at time-weighted average doses of 20 ng/kg body weight/day or 20 μg/kg body weight/day. The lower level was exposure-informed; the higher level served as a toxicological contrast. Glucose tolerance, systemic insulin responsiveness, and hepatic and whole-pancreas biochemical, histological, transcriptomic, and protein endpoints were evaluated. All three compounds altered glucose homeostasis, with overlapping but compound-dependent effects. Hepatic measurements showed lower insulin-signaling and glycogen-storage markers, including lower post-insulin AKT phosphorylation. Whole-pancreas measurements showed lower ESRRG and higher LDHA abundance, generating hypotheses about metabolic and secretion-associated pathways. Across the measured endpoints, CDP and RDP did not show a consistently smaller metabolic response than TPhP. Thus, their substitution cannot be presumed to improve metabolic safety. This comparison is restricted to the tested nominal doses and intermittent oral-gavage regimen; it does not establish overall toxicity or internal-dose-normalized relative potency. Internal dosimetry and comparisons with continuous exposure are needed to assess the relevance to human exposure.

## 1. Introduction

Organophosphate flame retardants (OPFRs) have been increasingly used as replacements for polybrominated diphenyl ethers in electronics, furniture, building materials, textiles, and vehicle interiors. Because most OPFRs are incorporated as additives rather than covalently bound components, they can migrate from consumer products throughout manufacturing, use, abrasion, and disposal. Their expanding production, widespread detection, and incomplete toxicological characterization have raised concern that the transition from brominated flame retardants may represent a regrettable substitution [1,2,3,4]. Human exposure occurs through ingestion of contaminated food and dust, inhalation, and dermal contact, while internal exposure has been documented in blood, urine, placenta, and other biological matrices [3,4].

Aryl OPFRs are of particular concern in metabolic toxicology because their relatively high hydrophobicity promotes interactions with lipid membranes and may favor partitioning into lipid-rich biological compartments [5]. Triphenyl phosphate (TPhP) is a high-production aryl OPFR for which experimental evidence increasingly links exposure to hepatic insulin resistance, altered adiponectin signaling, endoplasmic reticulum stress, and impaired carbohydrate metabolism [6,7]. Cresyl diphenyl phosphate (CDP) and resorcinol bis(diphenyl phosphate) (RDP) are commonly used alternatives with alkyl-substituted or oligomeric structures. CDP can directly engage liver X receptor α and promote SREBP-1c-dependent lipogenesis, and it also shows adipogenic activity through PPARγ-related mechanisms [8,9]. RDP undergoes extensive hepatic biotransformation and has been associated with mitochondrial dysfunction, oxidative stress, and broad metabolic disruption in aquatic models [10,11]. These observations challenge the assumption that larger molecular size and lower volatility necessarily confer improved biological safety.

Glucose homeostasis depends on coordinated insulin secretion by pancreatic β cells and insulin action in the liver and peripheral tissues [12,13]. The liver stores glucose after feeding and supports endogenous glucose production during fasting [14], whereas β cells couple glucose entry and metabolism to ATP generation, ATP-sensitive K^+^ (KATP)-channel closure, membrane depolarization, Ca^2+^ influx, and insulin-granule exocytosis [12,15]. Previous work has primarily examined either hepatic endpoints or pancreatic cell models in isolation. TPhP has been shown to alter β-cell homeostasis and glucose-responsive functions before overt cytotoxicity, suggesting that metabolic impairment may precede substantial tissue loss [16]. OPFR mixtures can also alter energy homeostasis in an estrogen receptor α-dependent and sex-dependent manner [17]. Epidemiological and experimental studies collectively support an association between OPFR exposure and metabolic dysfunction, but direct comparative evidence for TPhP and its current alternatives remains limited [18].

Low-dose endocrine and metabolic toxicants may exhibit non-monotonic responses because receptor occupancy, feedback regulation, xenobiotic metabolism, adaptive stress responses, and overt toxicity differ across exposure ranges [19]. Consequently, risk evaluation based only on high-dose monotonic assumptions may overlook biologically relevant responses near environmental exposure levels. Comparative studies that integrate systemic physiology with tissue-specific molecular endpoints are therefore needed to identify shared and compound-specific mechanisms.

In this study, male C57BL/6J mice were exposed orally for 70 days to TPhP, CDP, or RDP at an exposure-informed lower dose and a three-orders-of-magnitude higher experimental-contrast dose. Oral glucose tolerance, systemic insulin responsiveness, and glucose-stimulated insulin responses were combined with liver and whole-pancreas biochemistry, histopathology, RNA sequencing, qRT-PCR, and Western blotting. We tested whether the compounds produce convergent systemic and tissue-level changes consistent with altered hepatic insulin signaling and pancreatic metabolic and secretion-associated pathways, while distinguishing measured endpoints from proposed mechanistic links.

## 2. Materials and Methods

### 2.1. Chemicals and Reagents

TPhP, CDP, and RDP standards (purity ≥98%) were obtained from Dr. Ehrenstorfer GmbH (Augsburg, Germany), and corn oil was used as the oral dosing vehicle. The physicochemical properties of the three compounds are summarized in Table S1. Commercial 96-well mouse ELISA kits from Shanghai Enzyme-linked Biotechnology Co., Ltd. (Shanghai, China) were used to determine hepatic glycogen (catalog no. ml037785), insulin receptor (IR; ml058257), insulin receptor substrate 1 (IRS-1; ml058448), GLUT1 (ml037552), and GLUT4 (ml037551); pancreatic GLUT1 (ml037552) and muscarinic acetylcholine receptor (M-AChR; ml037816); serum insulin (ml001983); and plasma alanine aminotransferase (ALT; ml063179) and aspartate aminotransferase (AST; ml058659). ATP in liver and pancreas was measured using an Enhanced ATP Assay Kit (S0027; Beyotime Biotechnology, Shanghai, China). Complete assay-kit information is provided in Table S2.

Primary antibodies used for immunoblotting were total AKT (ProMabio, catalog no. P17171; 1:5000), phospho-AKT (Ser473) (Proteintech, 28731-1-AP; 1:1000), phospho-AKT (Thr308) (Proteintech, 29163-1-AP; 1:1000), GYS2 (Proteintech, 22371-1-AP; 1:2000), GLUT4 (Abcam, ab216661; 1:1000), ESRRG (Invitrogen, PA5-27755; 1:1000), LDHA (Proteintech, 19987-1-AP; 1:2000), and HRP-conjugated β-actin (Proteintech, HRP-60008; 1:5000). The antibody list is also summarized in Table S3.

### 2.2. Animals and Exposure Design

Male C57BL/6J mice aged 6–8 weeks and weighing approximately 20 g were obtained from Shanghai SLAC Laboratory Animal Co., Ltd. Mice were maintained in a barrier facility at 25 ± 2 °C and 50–60% relative humidity under a 12 h light/12 h dark cycle, with ad libitum access to food and water. After a 7-day acclimation period, animals were randomly assigned by body weight to seven groups (Table 1): Control, TPhP-L, TPhP-H, CDP-L, CDP-H, RDP-L, and RDP-H (at least six mice per group).

The lower time-weighted average dose of 20 ng/kg body weight/day was selected as an exposure-informed experimental level rather than as a literal reproduction of human exposure. Published estimates place total dietary OPFR intake at 6–49 ng/kg body weight/day in a Swedish market-basket study and 44.3 ng/kg body weight/day in Chinese adults, while median adult intake of total tri-OPEs through house-dust ingestion was 8.2 ng/kg body weight/day [20,21,22]. Thus, 20 ng/kg body weight/day lies within the order of magnitude reported for total external OPFR intake; these mixture- and route-specific estimates do not establish compound-specific equivalence for TPhP, CDP, or RDP. The 20 μg/kg body weight/day level was selected as a three-orders-of-magnitude experimental contrast. It should not be interpreted as representative of typical environmental exposure; however, it is of the same order of magnitude as the 9.19 μg/kg body weight/day dose used in a recent 28-week murine TPhP study [23], providing in vivo precedent for examining effects in the low-microgram-per-kilogram range. Because analogous in vivo dose information for CDP and RDP remains limited, the same nominal level was applied across all three compounds to enable a controlled comparison rather than to imply equivalent environmental or internal exposure. Test compounds were prepared in corn oil and administered by oral gavage every 3 days for 70 days at 10 mL/kg body weight. Each gavage delivered 60 ng/kg body weight in the lower-level groups or 60 μg/kg body weight in the higher-level groups. Averaged over the 3-day dosing interval, these amounts correspond to 20 ng/kg body weight/day and 20 μg/kg body weight/day, respectively; they are not daily administrations. Intermittent bolus gavage differs from continuous dietary, dust, inhalation, and dermal exposure and may produce different absorption and concentration–time profiles. Control mice received an equivalent volume of corn oil, and dosing volumes were adjusted using the most recent body weight.

Clinical condition, activity, fur appearance, food and water intake, and overt adverse responses were observed throughout exposure. Body weight was recorded every 3 days. Metabolic tests were separated by at least 3 days to minimize carryover effects from fasting, glucose loading, or exogenous insulin. The animal protocol was approved by the Experimental Animal Ethics Committee of Tongji University (approval no. TJAD00225101).

### 2.3. Glucose Tolerance, Insulin Tolerance, and Glucose-Stimulated Insulin Response

For the oral glucose tolerance test (OGTT), mice were fasted for 16 h with free access to water. Baseline tail-vein glucose was measured using an Accu-Chek Active glucometer, followed by oral administration of 20% glucose at 2 g/kg body weight. Blood glucose was measured at 15, 30, 60, 90, and 120 min. For the insulin tolerance test (ITT), mice were fasted for 4 h, injected intraperitoneally with recombinant human insulin at 0.5 U/kg body weight, and monitored at 15, 30, 45, 60, 90, and 120 min. These glucose and insulin challenge doses have been used for metabolic phenotyping in mice [24]. Areas under the curve (AUCs) were calculated using the trapezoidal rule.

For glucose-stimulated insulin assessment, mice were fasted for 16 h. Blood was collected immediately before glucose administration and 30 min after oral glucose loading at 2 g/kg body weight. Serum was isolated after clotting and centrifugation, and insulin concentrations were determined using a mouse insulin ELISA kit (catalog no. ml001983; Table S2).

### 2.4. Tissue Collection and Biochemical Measurements

All six mice per group underwent metabolic testing. After completion of those tests, three mice per group were allocated to molecular analyses, and the remaining three mice per group were used for histopathology, biochemical assays, and Western blotting. qRT-PCR used three animals per group; the available RNA-sequencing dataset sizes are specified by tissue in Section 2.6. The metabolic and tissue analyses therefore involved different analytical replicate sets, and different tissue endpoints were not necessarily measured in the same individual animals. At the terminal time point, the adult C57BL/6J mice (approximately 20 g at enrollment; non-neonatal and well below 200 g) were euthanized by manual cervical dislocation. The procedure was performed only by personnel trained in and competent to carry out the technique. This physical method, which is acceptable with conditions for appropriately sized mice, was selected to avoid potential interference from chemical anesthetics with terminal biochemical, molecular, and transcriptomic measurements. The trained operator verified irreversible death in accordance with the approved protocol before any tissue was collected. The euthanasia procedure was included in the approved animal protocol (TJAD00225101), performed in accordance with the current Chinese national standard GB/T 39760-2021 (Laboratory Animal—Guidelines for Euthanasia) [25], and consistent with the AVMA Guidelines for the Euthanasia of Animals [26].

Serum and EDTA-anticoagulated plasma were aliquoted and stored at −80 °C until analysis. Frozen liver or pancreas was homogenized in pre-cooled phosphate-buffered saline or the kit-specified lysis buffer at a tissue-to-buffer ratio of approximately 1:9 (*w*/*v*), followed by centrifugation at 4 °C; the supernatant was collected, and total protein was determined by the BCA method. The glycogen, IR, IRS-1, GLUT1, GLUT4, M-AChR, insulin, ALT, and AST ELISAs were performed according to the corresponding kit instructions. Briefly, standards, samples, and blanks were loaded into the designated wells, incubated with the supplied detection reagents, washed thoroughly, developed with tetramethylbenzidine substrate, and terminated with stop solution. Absorbance was measured at 450 nm, and concentrations were calculated from kit-specific standard curves with correction for dilution. Tissue endpoints were normalized to total protein where applicable. Further methodological details are provided in Supplementary Text S1.

For ATP measurement, liver or pancreas was homogenized in pre-cooled ATP lysis buffer and centrifuged at approximately 12,000× *g* for 5 min at 4 °C. ATP detection working solution (100 μL per well) was added to a white 96-well plate and equilibrated for 3–5 min before 20 μL of ATP standard or sample was added. Luminescence was recorded immediately, ATP concentrations were calculated using the standard curve, and values were normalized to the corresponding total protein concentration.

### 2.5. Histological Analysis

Liver and pancreas samples fixed in 4% paraformaldehyde were dehydrated, paraffin-embedded, sectioned at approximately 4 μm, and stained with hematoxylin and eosin (H&E). Liver morphology was evaluated at 100× and 400× magnification. Neutral-lipid accumulation was assessed in 8–10 μm OCT-embedded liver cryosections stained with Oil Red O and imaged at 100× and 400× magnification. Images used for Oil Red O quantification were acquired under identical settings, group codes were masked during analysis, and the positive area was quantified in ImageJ (version 1.53a) by an investigator blinded to group allocation.

Pancreatic sections were examined descriptively at low and high magnification for acinar architecture, ducts, inflammation, necrosis, and islet morphology. The available fields did not provide a valid independent-animal stereological design; therefore, no field-level islet-cell counts are reported.

### 2.6. RNA Sequencing and Bioinformatic Analysis

Liver and pancreas tissues from the Control, TPhP-H, CDP-H, and RDP-H groups were analyzed by RNA sequencing. Three mice per group were allocated to RNA sequencing. The liver expression dataset contained three biological replicates per group. The available pancreatic expression files underlying the analyses shown contained three Control samples and two samples per exposed group. Total RNA was extracted and assessed for concentration, purity, and integrity. Libraries were prepared by mRNA enrichment, fragmentation, double-stranded cDNA synthesis, adapter ligation, size selection, and PCR amplification, followed by paired-end sequencing on an Illumina platform.

After adapter removal and low-quality read filtering, clean reads were aligned to the mouse reference genome and quantified at the gene level. Differential expression was analyzed using DESeq2 [27], and Benjamini–Hochberg-adjusted *p* values were calculated. The liver candidate lists and volcano plots used nominal (unadjusted) *p* < 0.05 and |log2 fold change| > 0.5. The pancreatic lists and volcano plots used adjusted *p* < 0.05 and |log2 fold change| > 1. These tissue-specific screening criteria were not interchangeable, and DEG counts were not used to rank tissue susceptibility or compound potency. Both small-sample expression datasets remain exploratory, and statistical significance alone does not establish a mechanism.

Pancreatic marker-gene PCA used 118 manually curated genes selected by established cell-identity or compartment-associated functions and availability in the expression matrix, independently of differential-expression results or group separation. The selection criteria and complete gene list are provided in Supplementary Text S3. TPM values were transformed as log_2_(TPM + 1), then centered and scaled to unit variance per gene. Both the marker-gene PCA and the overall-transcriptome PCA (Figure S1) retained all nine samples. These descriptive analyses assessed expression heterogeneity without correcting tissue-composition differences, estimating cell proportions, establishing β-cell specificity, or independently demonstrating treatment effects.

Functional enrichment was performed using clusterProfiler [28] against Kyoto Encyclopedia of Genes and Genomes pathways. Gene set enrichment analysis (GSEA) used all genes ranked by the DESeq2 Wald statistic, gene-set sizes of 10–500, and 1000 gene-set permutations rather than phenotype-label permutations; nominal *p* < 0.05 and false-discovery-rate q < 0.25 were used as exploratory pathway-level criteria [29]. Pathway findings were interpreted as hypothesis-generating in relation to qRT-PCR, protein, biochemical, and physiological observations.

### 2.7. Quantitative Real-Time PCR

Total RNA was isolated from 30–50 mg frozen tissue using TRNzol Universal. One microgram of RNA was treated to remove genomic DNA and reverse-transcribed. qPCR was performed in a 10 μL reaction system using the following program: 95 °C for 30 s, followed by 40 cycles of 95 °C for 5 s and 60 °C for 30 s, with melt-curve analysis. Each biological sample was analyzed in three technical wells. The three Ct values were averaged within each animal before statistical analysis; technical wells were not counted as independent biological replicates. Relative expression was calculated using the 2^−ΔΔCt^ method with *Actb* as the reference gene.

Hepatic validation targets included *Socs3*, *Ppp1r3b*, *Nos2*, *Srebf1*, *Pck1*, *Nfkbia*, *Tbc1d4* (encoding AS160), *Gys2*, and *Slc2a2*. Pancreatic targets included *Esrrg*, *Pclo*, *Sgk1*, *Slc19a2*, *Nmnat1*, *Ldha*, *Me1*, *Fasn*, and *Elovl6*. Mouse gene symbols are italicized with an initial capital letter (e.g., *Slc2a1* and *Slc2a4*), whereas protein products are presented in non-italic uppercase form (e.g., GLUT1 and GLUT4).

### 2.8. Western Blotting and Acute Insulin Stimulation

Frozen liver or pancreas was lysed in RIPA buffer containing protease and phosphatase inhibitors. Equal protein amounts were separated by SDS-PAGE, transferred to PVDF membranes, blocked, and incubated with primary antibodies followed by horseradish peroxidase-conjugated secondary antibodies. Signals were detected by enhanced chemiluminescence and quantified using ImageJ. Hepatic GYS2, GLUT4, total AKT, phospho-AKT (Thr308), and phospho-AKT (Ser473), and pancreatic ESRRG and LDHA were measured. GYS2, GLUT4, ESRRG, LDHA, and total AKT were normalized to β-actin, whereas phospho-AKT was normalized to total AKT.

To assess hepatic AKT phosphorylation after insulin, a subset of mice received an acute intraperitoneal injection of insulin (0.25 U/kg body weight), and liver samples were collected 30 min later. The design included unstimulated and insulin-stimulated Control groups, whereas each OPFR-exposed group was sampled only after insulin injection. Accordingly, comparisons with Control+INS show lower post-insulin phospho-AKT in exposed animals but cannot distinguish a change in basal phosphorylation from an attenuated response to insulin. Additional procedural details are provided in Supplementary Text S2.

### 2.9. Statistical Analysis

Data were analyzed using GraphPad Prism (version 8.0.1), SPSS (version 21), and R (version 4.6.1). The individual animal was the independent experimental unit. OGTT and ITT used *n* = 6 mice per group; liver RNA sequencing and qRT-PCR used *n* = 3 biological replicates per group; the pancreatic expression-file sample counts are specified in Section 2.6; and histology, biochemical assays, and Western blotting used a separate *n* = 3 mice per group. Technical wells or repeated fields were not treated as independent *n* values. No a priori power calculation was performed for the small tissue datasets; these analyses are therefore considered exploratory and their limited statistical power is acknowledged. Unless otherwise stated, data are presented as mean ± standard deviation. Normality and homogeneity of variance were evaluated using the Shapiro–Wilk and Levene tests. One-way analysis of variance followed by Dunnett or Tukey multiplicity-adjusted comparisons was used when parametric assumptions were met. Repeated-measures two-way analysis of variance was applied to time-course data. Non-parametric data were analyzed using the Kruskal–Wallis test with adjusted post hoc comparisons. *p* < 0.05 was considered statistically significant for non-omics endpoints; transcriptomic multiplicity and exploratory criteria are described in Section 2.6.

## 3. Results

### 3.1. Aryl OPFR Exposure Impaired Systemic Glucose Handling

During the OGTT, blood glucose concentrations peaked at 15–30 min in all groups and subsequently declined (Figure 1a–c). Several exposed groups showed higher peak values, delayed recovery, or both. OGTT AUC was elevated most clearly in TPhP-L, CDP-H, and RDP-H mice (Figure 1d), indicating reduced disposal of an acute glucose load. Glucose-stimulated serum insulin increased at 30 min in every group, showing that the pancreas retained a measurable response to acute glucose exposure; however, baseline and stimulated levels varied among treatments (Figure 1e).

After exogenous insulin administration, glucose decreased rapidly but recovered earlier or remained higher in several exposed groups (Figure 1g–i). ITT AUC was increased particularly in CDP-L and both RDP groups, with the strongest response in RDP-H mice (Figure 1f). ITT reflects whole-body insulin responsiveness and cannot by itself establish liver-specific insulin resistance. Differences between the two exposure levels suggest the possibility of a non-monotonic response for some endpoints; the dose–response shape cannot be established from two nonzero dose levels.

### 3.2. Exposure Was Associated with Hepatic and Whole-Pancreas Biochemical Changes

Biochemical measurements identified liver and whole-pancreas molecular changes associated with the systemic phenotype. Hepatic glycogen was reduced in all higher-level groups, with especially pronounced decreases after TPhP-H and RDP-H exposure (Figure 2a). IR and IRS-1 also declined, most clearly in the higher-level groups (Figure 2b,d), indicating reduced abundance of proximal insulin-signaling components. Hepatic ATP was reduced after higher-level exposure (Figure 2e). Hepatic GLUT1 and GLUT4 showed compound- and level-dependent changes, with the strongest suppression generally occurring in higher-level groups (Figure 2c,f). These *n* = 3 tissue measurements describe associated insulin signaling-related and energy-storage changes. They do not independently quantify hepatic insulin sensitivity or establish a causal pathway.

Whole-pancreas endpoints displayed compound-specific patterns. GLUT1 increased in TPhP-L mice but decreased sharply in TPhP-H mice, while RDP produced a progressive decline and RDP-H approached the lowest measured level (Figure 3a). M-AChR was reduced in the TPhP-H and RDP-H groups (Figure 3b), which may indicate altered cholinergic modulation but does not directly demonstrate impaired cholinergic amplification of insulin secretion. Whole-pancreas ATP was reduced in the TPhP-H and CDP-H groups and showed a downward tendency in RDP-H mice (Figure 3c). Because endocrine cells constitute a small fraction of pancreatic tissue, these measurements cannot be assigned specifically to β cells.

Plasma AST and ALT were elevated in most exposed groups, with larger changes generally observed at the higher level (Figure S2). H&E-stained liver sections showed largely preserved lobular architecture with mild hepatocellular swelling, irregular arrangement, or focal inflammatory changes in selected higher-level groups; 100× and 400× views are provided in Figures S3 and S4. Oil Red O staining showed increased neutral-lipid accumulation, with representative 100× and 400× images and blinded semiquantification provided in Figures S5–S7. Pancreatic histology did not show widespread necrosis, hemorrhage, or severe inflammation; representative high-magnification islet fields are shown descriptively in Figure S8. No field-level islet-cell counts are reported because the sampling structure did not support stable animal-level inference.

### 3.3. Liver Transcriptomics Identified Exploratory Insulin-Signaling, Glycogen, and Lipid-Metabolism Patterns

Higher-level TPhP, CDP, and RDP exposure produced exploratory hepatic transcriptional changes under the nominal-P screening criteria described in Section 2.6 (Figure 4a–c). Representative candidates included *Ppp1r3b*, *Serpine1*, *Jun*, *Brd2*, *Dhcr7*, and *Flvcr2*, encompassing genes related to hepatic glycogen handling, stress and matrix remodeling, chromatin-dependent metabolic regulation, cholesterol biosynthesis, and solute transport [30,31,32,33,34]. PCA separated Control samples from most exposed samples primarily along PC1, which explained 32.7% of total variance, whereas PC2 explained 15.7% (Figure 4d). The dispersion of one CDP replicate and partial overlap among exposure groups indicate substantial within-group heterogeneity; therefore, individual DEG and pathway findings are treated as hypothesis-generating.

Exploratory KEGG enrichment implicated insulin-signaling, PI3K-AKT, AMPK, FoxO, PPAR, glycolysis/gluconeogenesis, starch and sucrose metabolism, pyruvate metabolism, fatty-acid degradation, fatty-acid biosynthesis, and peroxisomal pathways (Figure 4e–g). Gene-set-permutation GSEA provided directional support for selected pathway patterns: TPhP showed negative enrichment of starch/sucrose metabolism, fatty-acid degradation, and PPAR signaling; CDP showed altered pentose-phosphate and fatty-acid-degradation programs; and RDP showed negative enrichment of peroxisomal and fatty-acid-degradation pathways (Figure S9). Given *n* = 3, these enrichments are exploratory and require independent functional confirmation.

qRT-PCR identified a shared hepatic expression pattern (Figure S10). *Socs3*, *Nos2*, *Nfkbia*, *Pck1*, and *Srebf1* were induced, while *Ppp1r3b*, *Tbc1d4*, *Gys2*, and *Slc2a2* showed lower expression, with statistical support varying among compounds. Together with the biochemical and protein endpoints, these changes suggest hypotheses involving inflammatory feedback, insulin signaling, glycogen handling, and lipid metabolism. The *n* = 3 expression measurements do not establish pathway activity or metabolic flux.

### 3.4. Whole-Pancreas Transcriptomics Identified Exploratory Changes in Metabolic and Secretion-Associated Programs

Using the pancreatic adjusted-*p* and fold-change criteria specified in Section 2.6, whole-pancreas RNA sequencing identified 498, 250, and 352 candidate genes after TPhP, CDP, and RDP exposure, respectively (Figure 5a–c). Representative downregulated candidates included *Pclo*, *Slc19a2*, *Nmnat1*, *Sgk1*, and *Esrrg*, whereas *Fasn*, *Snca*, *Alas2*, and other stress- or metabolism-associated genes were induced. PCA based on curated pancreatic marker genes separated Control samples from exposed samples mainly along PC2, which explained 20.7% of variance; PC1 explained 46.5% (Figure 5d). The small sample size and bulk-tissue composition make these findings hypothesis-generating.

Exploratory KEGG enrichment implicated PPAR signaling, glycolysis/gluconeogenesis, PI3K-AKT, FoxO, apoptosis, glucagon signaling, cAMP signaling, Ca^2+^ signaling, MAPK, mTOR, oxidative phosphorylation, and insulin secretion (Figure 5e–g). These gene sets are related to nutrient sensing, mitochondrial function, membrane excitability, and secretion, but enrichment does not demonstrate functional impairment of those processes. Gene-set-permutation GSEA identified pathway patterns involving protein secretion and E2F targets after TPhP, insulin-secretion and mTORC1 programs after CDP, and glycolysis and oxidative phosphorylation after RDP (Figure S11); these results are considered hypothesis-generating.

Whole-pancreas qRT-PCR further characterized the molecular pattern (Figure S12). *Esrrg*, *Pclo*, *Slc19a2*, *Nmnat1*, and *Sgk1* were generally reduced, whereas *Ldha*, *Me1*, *Fasn*, and *Elovl6* were induced. This combination indicates changes in oxidative-metabolism-, glucose-sensing-, and lipid-synthesis-associated programs. Metabolic flux and β-cell specificity remain unresolved.

### 3.5. Protein Analyses Identified Lower Hepatic Phospho-AKT After Insulin and Whole-Pancreas ESRRG–LDHA Changes

Western blotting characterized hepatic glycogen-related proteins, post-insulin AKT phosphorylation, and whole-pancreas ESRRG and LDHA abundance (Figure 6). Hepatic GYS2 decreased after exposure, with significant suppression in multiple CDP and RDP groups and the greatest reduction in RDP-H mice (Figure 6a,d). Hepatic GLUT4 showed a modest downward tendency but did not reach significance in most groups (Figure 6a,e), consistent with the liver relying primarily on GLUT2 for hepatocyte glucose transport.

Whole-pancreas ESRRG protein was reduced across all exposure groups, including the lower-level groups, whereas LDHA protein increased approximately nine- to ten-fold (Figure 6b,f,g). These changes were concordant with the qRT-PCR results and describe an association between lower ESRRG and greater LDHA abundance in bulk tissue. With *n* = 3 biological replicates per group, this pattern is hypothesis-generating. Its cellular origin and functional consequences remain unresolved because lactate production, pyruvate oxidation, oxygen consumption, NADH/NAD+ balance, and ATP/ADP ratio were not measured.

Total hepatic AKT remained near Control levels (Figure 6c,h). Insulin increased AKT phosphorylation at Thr308 and Ser473 in Control+INS mice, whereas exposed groups generally showed lower post-insulin phosphorylation (Figure 6i,j). At Ser473, the TPhP-H comparison with Control+INS did not reach statistical significance. The absence of matched unstimulated exposed groups prevents separation of basal phosphorylation from the response to insulin. These findings indicate insulin signaling-related changes, with insufficient evidence to establish hepatic insulin resistance directly.

## 4. Discussion

### 4.1. Systemic Functional Changes and Tissue-Level Molecular Associations

This study provides comparative in vivo evidence that TPhP, CDP, and RDP alter glucose homeostasis together with liver and whole-pancreas molecular endpoints. OGTT and ITT demonstrate altered systemic glucose disposal and insulin responsiveness, while the tissue measurements identify associated molecular and biochemical changes at the organ level. The relatively mild histopathological findings suggest that these changes occurred without widespread tissue destruction.

Differences between the lower- and higher-dose responses suggest the possibility of a non-monotonic response for some endpoints. Two nonzero dose levels are insufficient to establish the shape of a dose–response relationship. Feedback regulation, metabolism, and adaptation provide possible biological explanations [19], which require testing across additional dose levels.

### 4.2. Hepatic Insulin Signaling-Related Molecular Changes

The liver maintains glucose homeostasis through coordinated glycogen storage and endogenous glucose production [35]. Lower hepatic IR and IRS-1, reduced GYS2 and glycogen, increased *Pck1*, and lower post-insulin phospho-AKT were observed in this study. These associations identify insulin signaling-related changes within pathways relevant to hepatic glucose regulation [36,37]. They do not directly establish hepatic insulin resistance. ITT measures whole-body insulin responsiveness, and the absence of matched basal phospho-AKT measurements in exposed animals prevents quantification of the insulin-induced change in phosphorylation. Total AKT abundance was largely preserved.

Reduced hepatic ATP suggests altered energy status that could influence glucose phosphorylation, glycogen synthesis, transport, and signaling. Previous studies have reported disturbed hepatic carbohydrate metabolism after TPhP exposure and altered oxidative phosphorylation and lipid handling after OPFR exposure [38,39,40,41]. Mitochondrial respiration and metabolic flux remain to be evaluated directly; in the present study, ATP serves as a supportive biochemical indicator of altered cellular energetics.

Neutral-lipid accumulation was observed across compounds. *Srebf1* induction, exploratory PPAR/fatty-acid-degradation patterns, and increased Oil Red O staining suggest altered lipid handling. The CDP profile is compatible with the LXRα–SREBP-1c-related effects reported previously [8,42], whereas RDP showed negative enrichment of peroxisomal and fatty-acid-degradation gene sets. These *n* = 3 tissue observations generate hypotheses about lipid regulation; statistical significance does not establish altered lipid flux or an underlying causal mechanism.

Inflammatory and nitrosative signaling may have further amplified this metabolic disturbance. *Socs3*, *Nos2*, and *Nfkbia* were induced, accompanied by increased plasma aminotransferase activities and focal inflammatory changes. SOCS3 can interfere with IR–IRS signaling by reducing IRS tyrosine phosphorylation, whereas excessive iNOS-derived NO and peroxynitrite can impair mitochondrial function and modify IR, IRS-1, and AKT through S-nitrosylation or tyrosine nitration [43,44,45]. Tissue NO production, protein nitration, and S-nitrosylation were not measured, so these pathways remain plausible contributors to the observed hepatic profile. Figure 7 integrates the associated hepatic endpoints.

### 4.3. Whole-Pancreas ESRRG–LDHA Associations Generate Hypotheses About Metabolic and Secretion-Related Programs

Glucose-stimulated insulin secretion depends on glucose metabolism, ATP generation, ion-channel activity, Ca^2+^ entry, and granule fusion [46,47]. In whole pancreas, GLUT1, ATP, M-AChR, and transcriptomic programs related to oxidative phosphorylation, Ca^2+^, cAMP, insulin secretion, and protein secretion changed in selected groups. Reduced M-AChR is compatible with altered cholinergic modulation, although receptor abundance alone does not quantify cholinergic amplification. Because these measurements were obtained from bulk pancreas, their cellular origin remains unresolved.

ESRRG was reduced in whole pancreas. ERRγ has established roles in the metabolic maturation of glucose-responsive β cells and in pancreatic acinar-cell metabolism [48,49]. In this study, lower *Esrrg*/ESRRG, *Slc19a2*, and *Nmnat1* support a hypothesis involving oxidative- and maturation-associated programs [50,51]. Because these are bulk-tissue measurements, the pattern cannot identify the contributing cell populations or establish a β-cell-specific causal relationship. Whole-pancreas ATP likewise reflects both endocrine and exocrine compartments.

LDHA induction provides a complementary observation. In β-cell models, forced LDHA expression can weaken glucose-induced insulin secretion by changing pyruvate handling [52,53]. The approximately nine- to ten-fold increase in whole-pancreas LDHA, together with *Ldha* and *Me1* induction, raises a hypothesis about altered pyruvate and NADPH metabolism. It does not demonstrate increased lactate production or a causal link between ESRRG reduction and LDHA induction. Lactate production, pyruvate oxidation, NADH/NAD+ balance, and metabolic flux remain unmeasured. *Fasn* and *Elovl6* induction similarly identifies a lipid-synthesis-associated expression pattern [54,55].

*Pclo* and secretion-associated gene sets also changed. Piccolo participates in Ca^2+^- and cAMP-responsive exocytotic machinery [56,57], providing biological context for the observed expression pattern. Whether these bulk-tissue associations correspond to altered β-cell secretion requires isolated-islet functional assays, granule-dynamics measurements, and cell-specific perturbation of ESRRG and LDHA. Figure 8 summarizes the observations and proposed links as a hypothesis-generating model.

### 4.4. Shared and Compound-Specific Profiles Across Selected Metabolic Endpoints

All three aryl OPFRs altered systemic glucose homeostasis and selected tissue endpoints, but their response profiles differed. TPhP produced broad exploratory whole-pancreas transcriptional changes; CDP showed a relatively lipogenic pattern; and RDP produced pronounced changes in systemic insulin responsiveness, hepatic IR/IRS-1–AKT-related markers, fatty-acid-degradation gene sets, and whole-pancreas GLUT1, M-AChR, ESRRG, and LDHA. These differences may reflect distinct absorption, distribution, metabolism, receptor interactions, and internal concentrations, none of which were quantified here. Previous mouse studies have also reported metabolic responses that varied with diet, sex, dose, and developmental exposure window [58,59,60].

CDP and RDP cannot be presumed metabolically safer than TPhP on the basis of their replacement status. Across the measured endpoints, neither alternative showed a consistently smaller response under the tested nominal doses and intermittent oral-gavage regimen (Table S4). Overall toxicity and internal-dose-normalized relative potency remain unresolved because serum and tissue concentrations were not measured and the compounds differ in physicochemical properties and predicted bioaccumulation. Replacement status, lower volatility, or greater molecular size alone therefore cannot be assumed to confer lower metabolic activity.

### 4.5. Strengths and Limitations

A strength of this work is the integration of systemic physiology with parallel liver and whole-pancreas measurements under one exposure design. The 20 ng/kg body weight/day time-weighted average is of the same order of magnitude as published total external OPFR-intake estimates, without reconstructing compound-specific human intake. The 20 μg/kg body weight/day level provided a toxicological contrast, with dose-range precedent from a long-term murine TPhP study [23]; it was not intended to simulate typical environmental exposure.

Intermittent bolus gavage every 3 days can produce a different concentration–time profile from continuous low-level exposure even when their nominal time-weighted doses are equal [61,62]. A bolus may produce higher transient concentrations followed by lower concentrations between administrations, depending on absorption and elimination. Metabolic responses could therefore reflect transient peak exposure, repeated recovery periods, or accumulation [62,63]. Without toxicokinetic measurements, the direction and magnitude of these effects cannot be determined, and neither internal exposure nor toxicity can be assumed equivalent to continuous human exposure [62]. The comparative findings consequently apply to the tested doses and oral-gavage schedule.

The absence of matched serum or tissue concentrations is a central limitation: equal nominal doses do not imply equal internal exposure, and relative potency cannot be established. No a priori power calculation was performed for the *n* = 3 tissue datasets; transcriptomic candidate lists based on nominal *p* values, GSEA results, histology, biochemical assays, and Western blots are exploratory and sensitive to inter-individual variation. Bulk-pancreas measurements cannot be assigned to β cells because endocrine tissue represents only a small fraction of the organ. The AKT design lacked basal exposed groups; the 0.5 U/kg ITT challenge may elicit a strong systemic response that reduces sensitivity to subtler group differences; and additional dose levels are required to characterize dose–response relationships. Finally, isolated-islet secretion, ATP/ADP ratio, oxygen consumption, lactate release, Ca^2+^ dynamics, KATP/voltage-dependent Ca^2+^ currents, hepatic glucose-production flux, and hyperinsulinemic–euglycemic clamp measurements were not performed. Accordingly, conclusions are limited to associations among systemic phenotypes and tissue-level molecular changes.

Future studies should combine continuous low-level dietary exposure with serial measurements of the parent compounds and relevant metabolites in blood and target tissues. Sampling across dosing intervals would help characterize peak concentrations, accumulation, and overall internal exposure. Larger prespecified sample sizes, additional dose levels, matched basal and insulin-stimulated liver sampling, isolated-islet functional assays, and cell-resolved pancreatic analyses are also needed to evaluate internal-dose-normalized relative potency, cellular origin, and causal sequence.

## 5. Conclusions

CDP and RDP cannot be presumed metabolically safer than TPhP for the glucose-metabolic endpoints evaluated in this study. Under the tested nominal doses and 70-day intermittent oral-gavage regimen, all three compounds altered systemic glucose regulation and produced hepatic and whole-pancreas molecular changes. The alternatives did not show consistently smaller responses across the measured endpoints. Hepatic findings identify insulin signaling-related and glycogen-storage changes, while whole-pancreas ESRRG–LDHA and secretion-associated patterns remain hypothesis-generating. These observations do not establish hepatic insulin resistance, β-cell-specific causality, overall toxicity equivalence, or internal-dose-normalized relative potency. Comparative internal dosimetry, continuous-exposure studies, broader dose–response evaluation, and direct functional testing are needed to assess substitution safety.

## Figures and Tables

**Figure 1:**
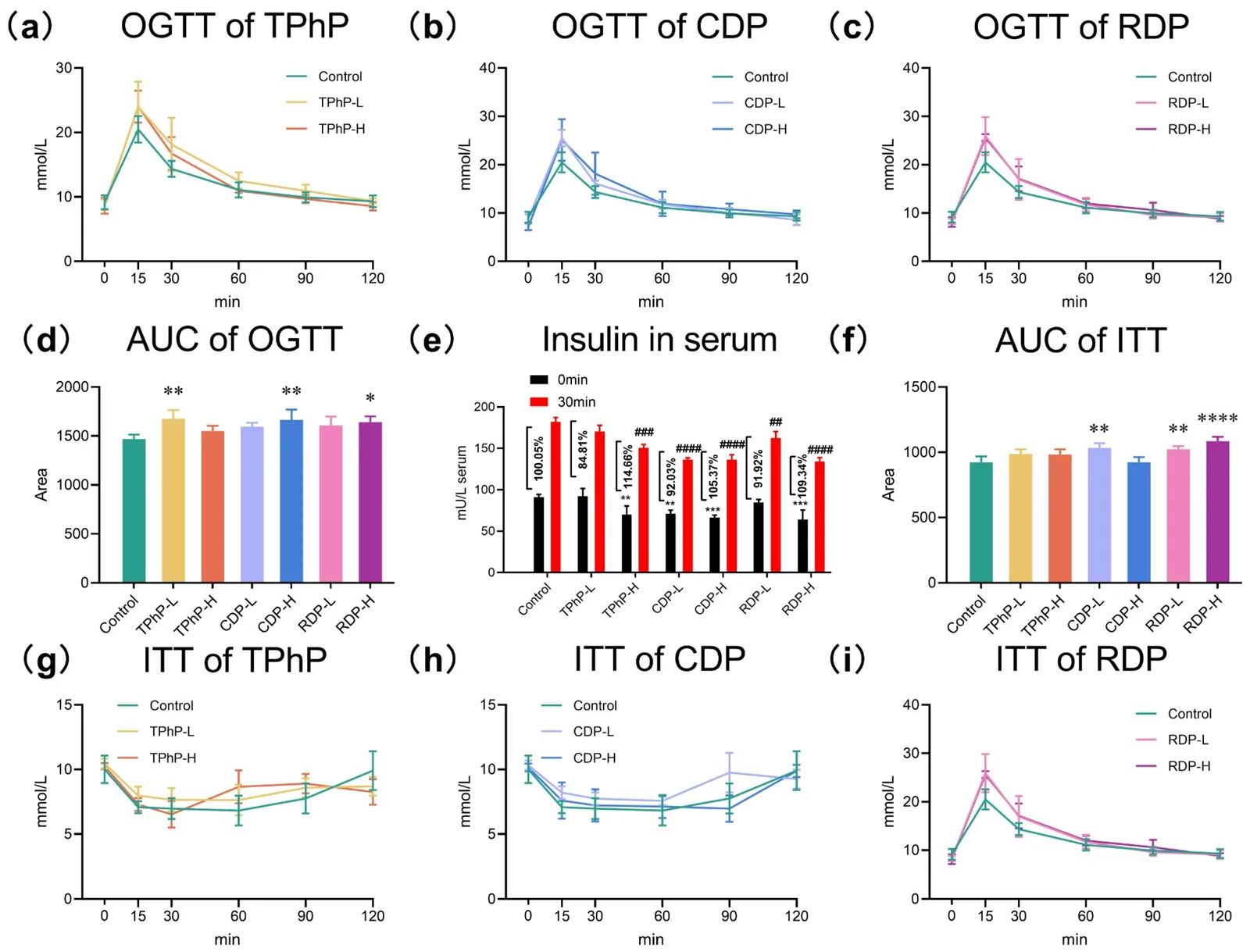
Effects of TPhP, CDP, and RDP exposure on glucose-metabolic phenotypes in mice. (**a**–**c**) Blood glucose curves during the OGTT; (**d**) OGTT area under the curve; (**e**) serum insulin concentrations before and 30 min after glucose loading; (**f**) ITT area under the curve; and (**g**–**i**) blood glucose curves during the ITT. Data are mean ± SD (*n* = 6 independent animals per group). ITT reflects whole-body insulin responsiveness and is not liver-specific. * *p* < 0.05, ** *p* < 0.01, *** *p* < 0.001, and **** *p* < 0.0001 versus Control in panels (**d**,**f**); ## *p* < 0.01, ### *p* < 0.001, and #### *p* < 0.0001 for comparisons between corresponding lower- and higher-level groups in panel (**e**); ns, not significant.

**Figure 2:**
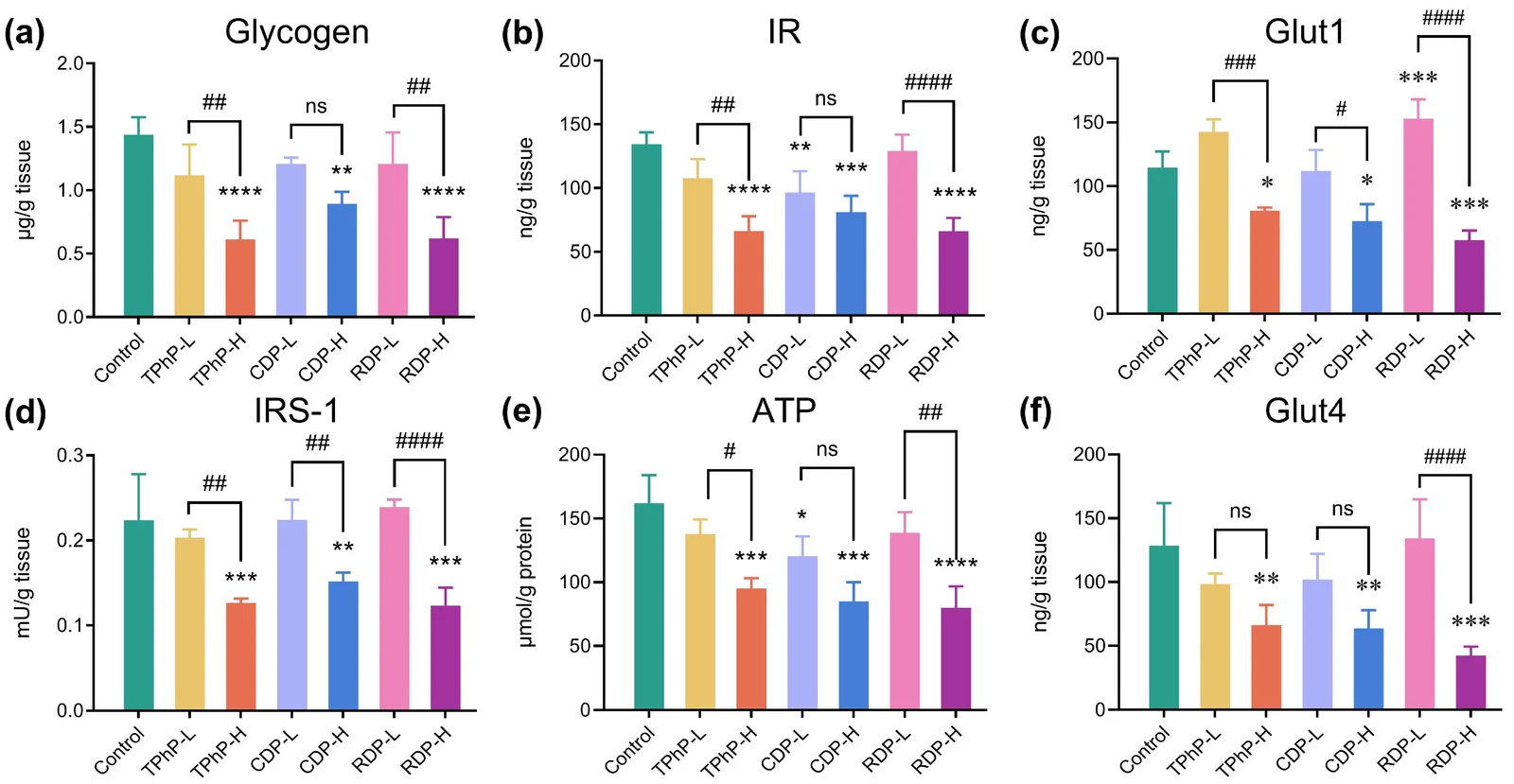
Effects of TPhP, CDP, and RDP exposure on hepatic endpoints related to glucose metabolism. (**a**) Glycogen; (**b**) insulin receptor (IR); (**c**) GLUT1; (**d**) IRS-1; (**e**) ATP; and (**f**) GLUT4. Data are mean ± SD (*n* = 3 independent animals per group; technical measurements were averaged within each animal). These animals constituted the tissue-analysis subset and were not an identical analytical replicate set to the *n* = 6 metabolic dataset. * *p* < 0.05, ** *p* < 0.01, *** *p* < 0.001, and **** *p* < 0.0001 versus Control; # *p* < 0.05, ## *p* < 0.01, ### *p* < 0.001, and #### *p* < 0.0001 between corresponding lower- and higher-level groups; ns, not significant.

**Figure 3:**
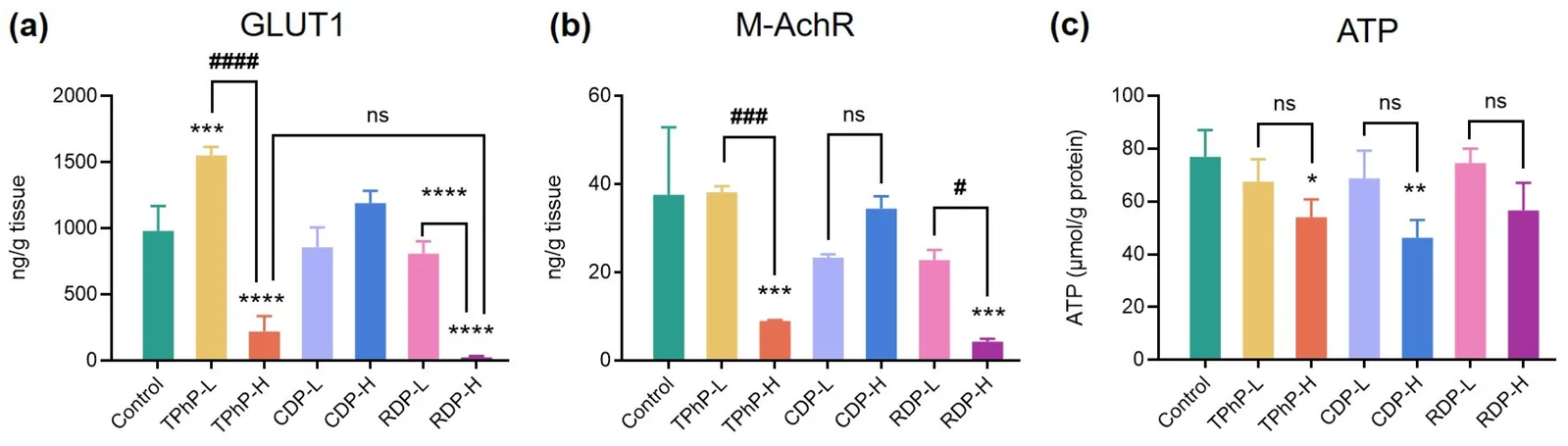
Effects of TPhP, CDP, and RDP exposure on whole-pancreas endpoints related to glucose metabolism. (**a**) GLUT1; (**b**) muscarinic acetylcholine receptor (M-AChR); and (**c**) ATP. Data are mean ± SD (*n* = 3 independent animals per group; technical measurements were averaged within each animal). These bulk-tissue measurements are not β-cell-specific. * *p* < 0.05, ** *p* < 0.01, *** *p* < 0.001, and **** *p* < 0.0001 versus Control; # *p* < 0.05, ### *p* < 0.001, and #### *p* < 0.0001 between corresponding lower- and higher-level groups; ns, not significant.

**Figure 4:**
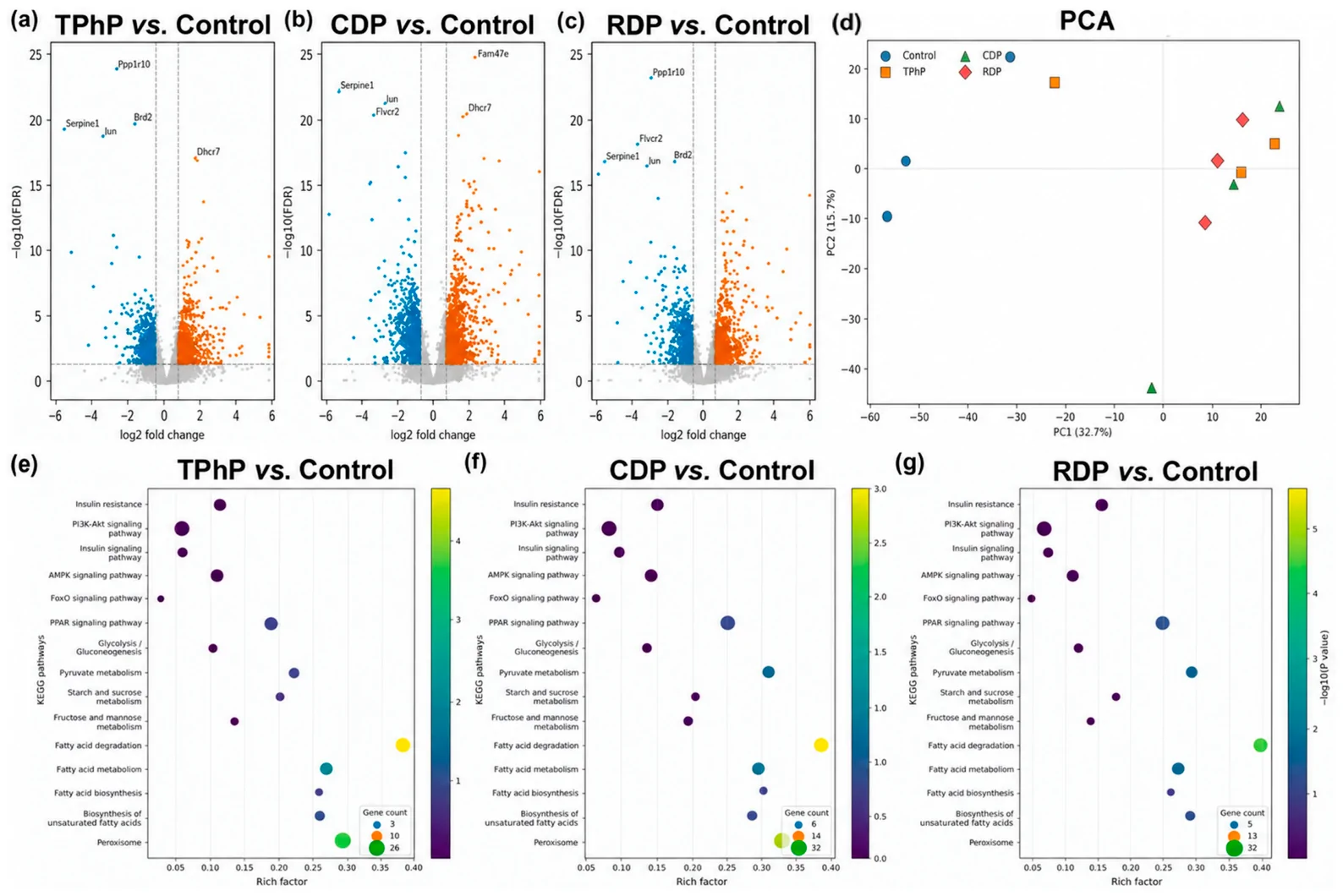
Exploratory liver transcriptomic alterations after higher-level TPhP, CDP, or RDP exposure. (**a**–**c**) Volcano plots relative to Control. Orange and blue dots denote genes meeting the exploratory screening criteria of nominal (unadjusted) *p* < 0.05 and |log2 fold change| > 0.5; gray dots do not meet both criteria. (**d**) PCA of hepatic transcriptomes; PC1 and PC2 explain 32.7% and 15.7% of variance. Each symbol is one biological replicate (*n* = 3 per group). (**e**–**g**) Exploratory KEGG enrichment analyses; bubble size indicates gene count and color indicates enrichment significance. Small *n* and the dispersed CDP replicate limit confirmatory interpretation.

**Figure 5:**
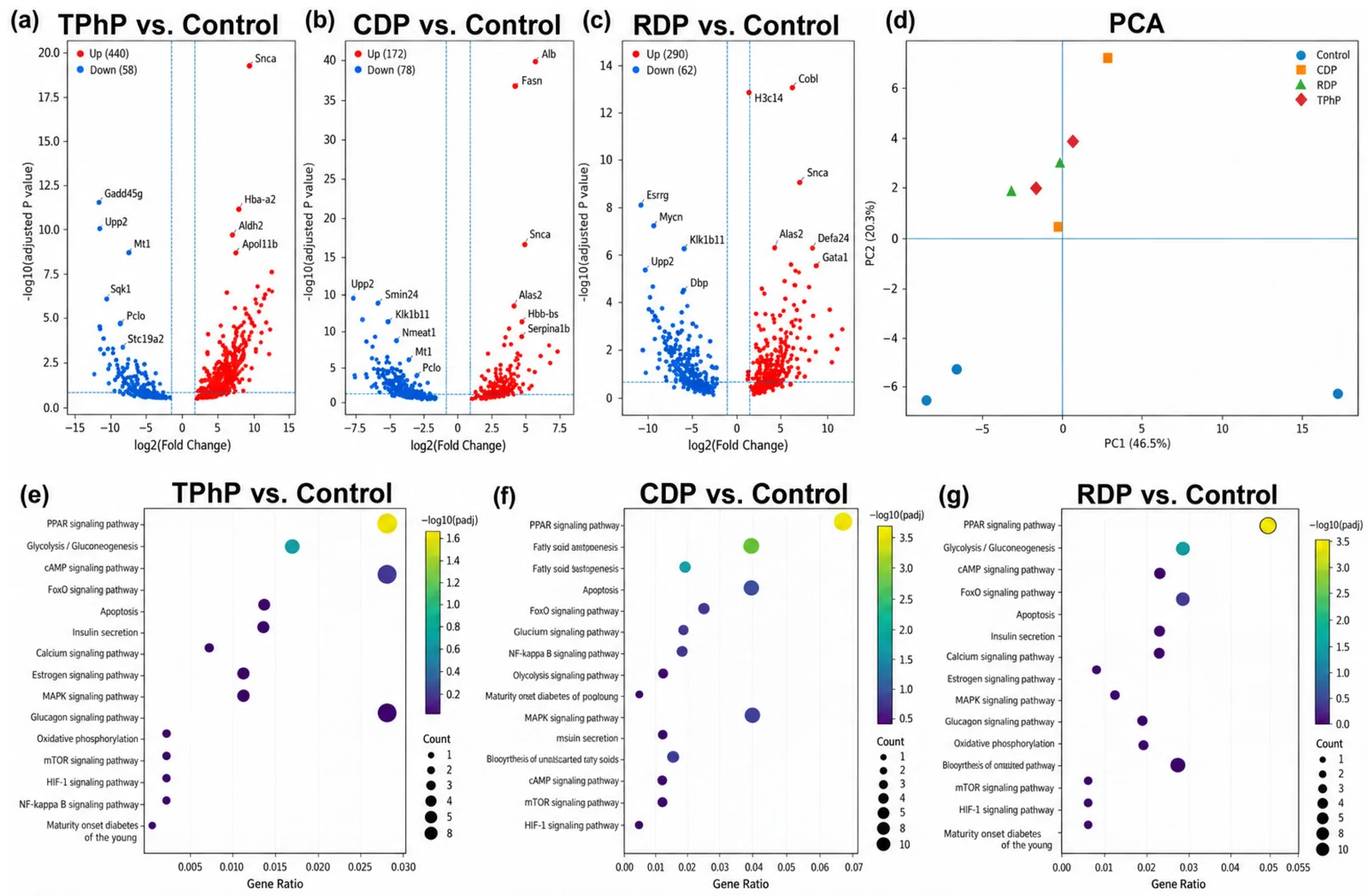
Exploratory whole-pancreas transcriptomic alterations after higher-level TPhP, CDP, or RDP exposure. (**a**–**c**) Volcano plots relative to Control. Red and blue dots denote genes meeting the exploratory screening criteria of Benjamini–Hochberg-adjusted *p* < 0.05 and |log2 fold change| > 1. (**d**) PCA based on 118 curated pancreatic cell-marker genes, as detailed in Section 2.6; PC1 and PC2 account for 46.5% and 20.7% of variance. Each symbol is one biological replicate (Control, *n* = 3; each exposed group, *n* = 2). (**e**–**g**) Exploratory KEGG pathway enrichment analyses. Bulk-tissue composition and small *n* limit β-cell-specific or confirmatory interpretation.

**Figure 6:**
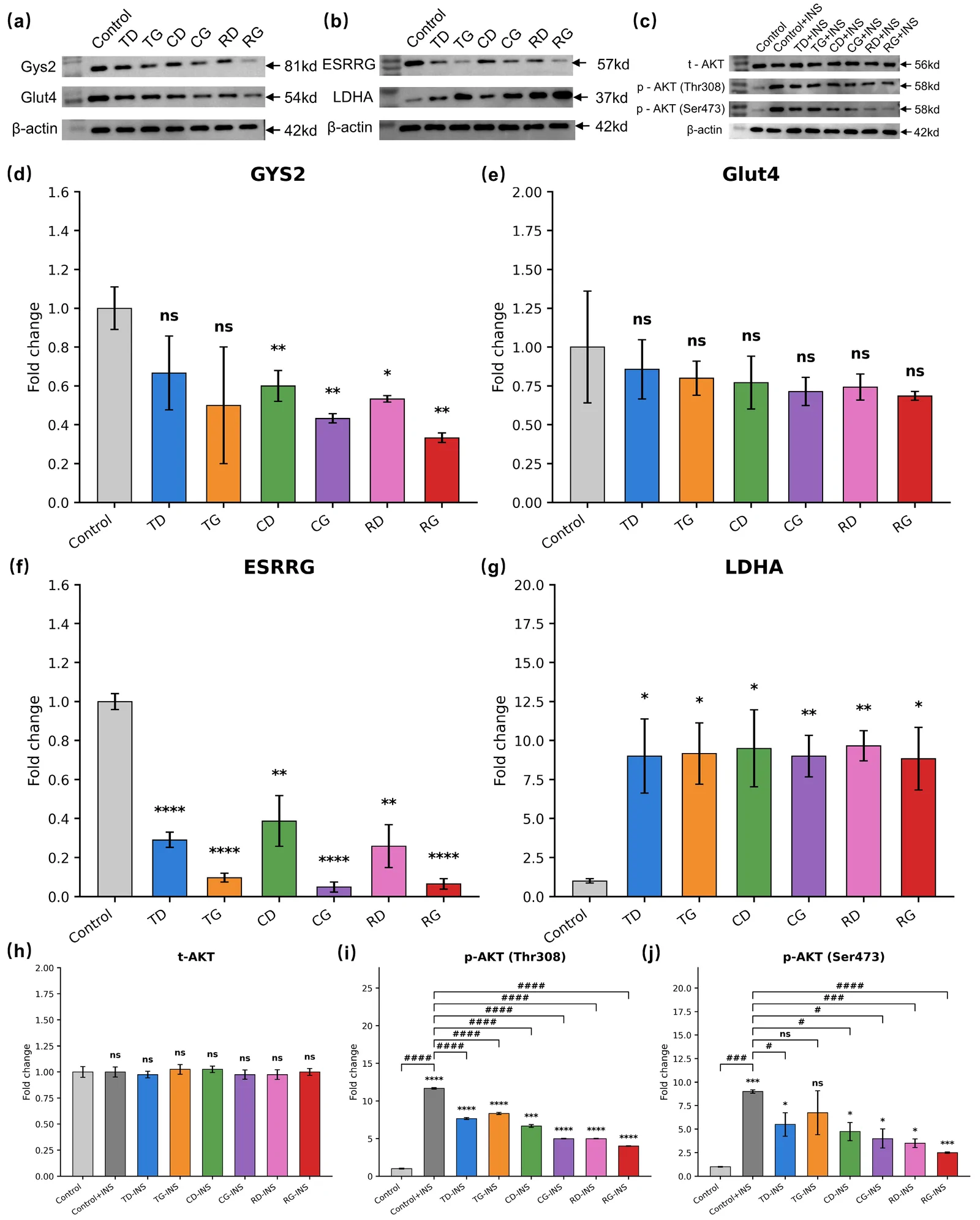
Effects of TPhP, CDP, and RDP exposure on hepatic and whole-pancreas proteins. (**a**) Hepatic GYS2 and GLUT4; (**b**) whole-pancreas ESRRG and LDHA; (**c**) hepatic total AKT (t-AKT), phospho-AKT (Thr308), and phospho-AKT (Ser473). (**d**–**g**) GYS2, GLUT4, ESRRG, and LDHA quantification; (**h**–**j**) t-AKT and phospho-AKT quantification. Data are mean ± SD (*n* = 3 independent animals per group; technical measurements were averaged within each animal). Exposed groups were sampled after insulin and compared with Control+INS; no basal exposed groups were included, so basal phosphorylation and insulin responsiveness cannot be separated. INS, insulin; * *p* < 0.05, ** *p* < 0.01, *** *p* < 0.001, and **** *p* < 0.0001 versus the corresponding Control; # *p* < 0.05, ### *p* < 0.001, and #### *p* < 0.0001 for the bracketed comparisons with Control+INS in panels (**i**,**j**); TD/TG, TPhP lower/higher level; CD/CG, CDP lower/higher level; RD/RG, RDP lower/higher level; ns, not significant.

**Figure 7:**
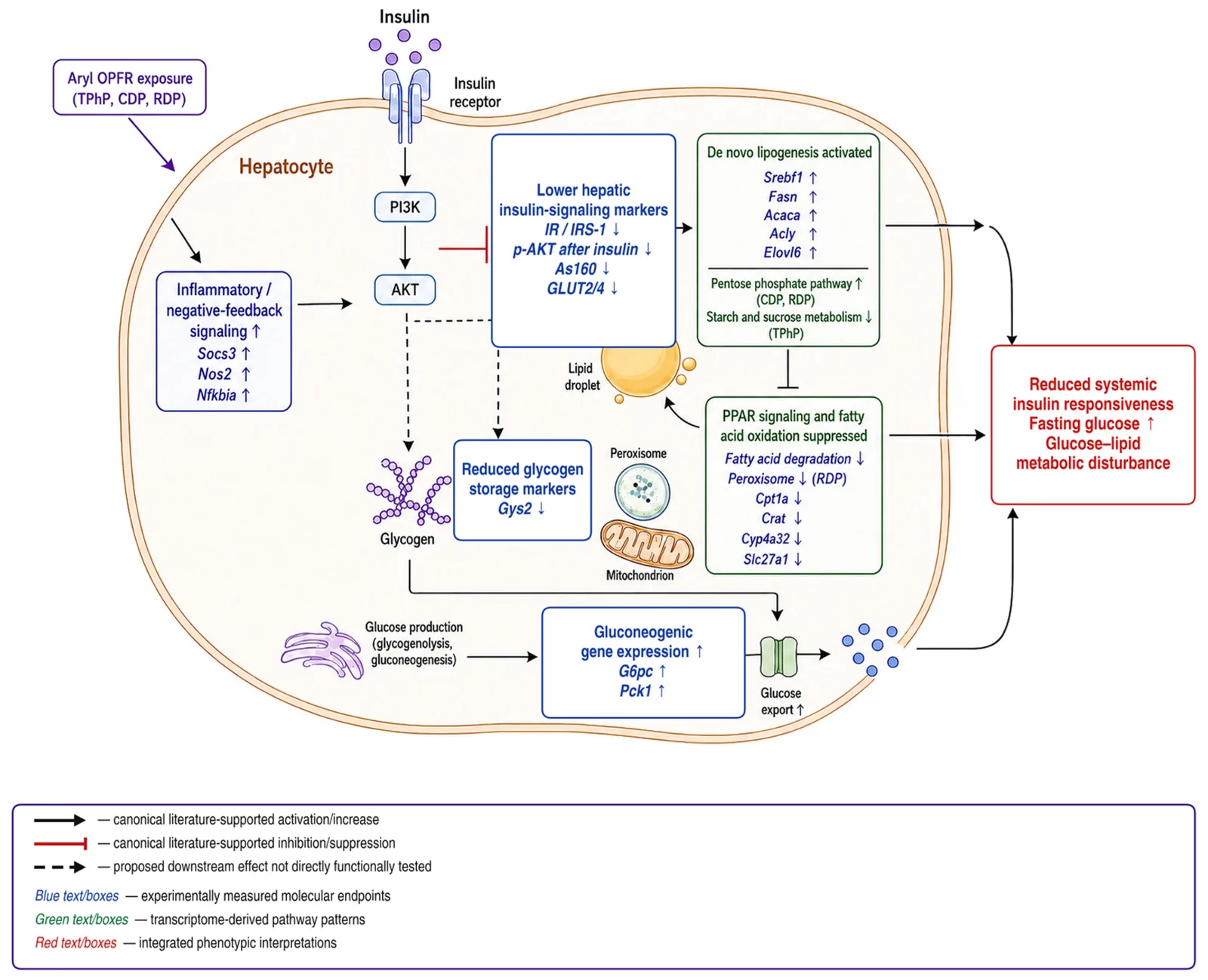
Evidence-integrated model of hepatic glucose- and lipid-metabolism changes associated with TPhP, CDP, and RDP exposure. Blue labels/boxes denote experimentally measured molecular endpoints; green labels/boxes denote transcriptome-derived pathway patterns; red labels/boxes denote integrated phenotypic interpretations. Solid arrows show canonical literature-supported relationships, whereas dashed arrows show proposed downstream effects that were not directly functionally tested. T-ended lines denote inhibition or suppression; upward and downward arrows beside labels indicate increases and decreases, respectively. Purple circles and the branched purple structure represent insulin and glycogen, respectively; blue circles represent glucose. Labeled organelle and lipid-droplet icons are schematic, and boxes group endpoints or interpretations. The model does not establish liver-specific insulin resistance or internal-dose-normalized potency.

**Figure 8:**
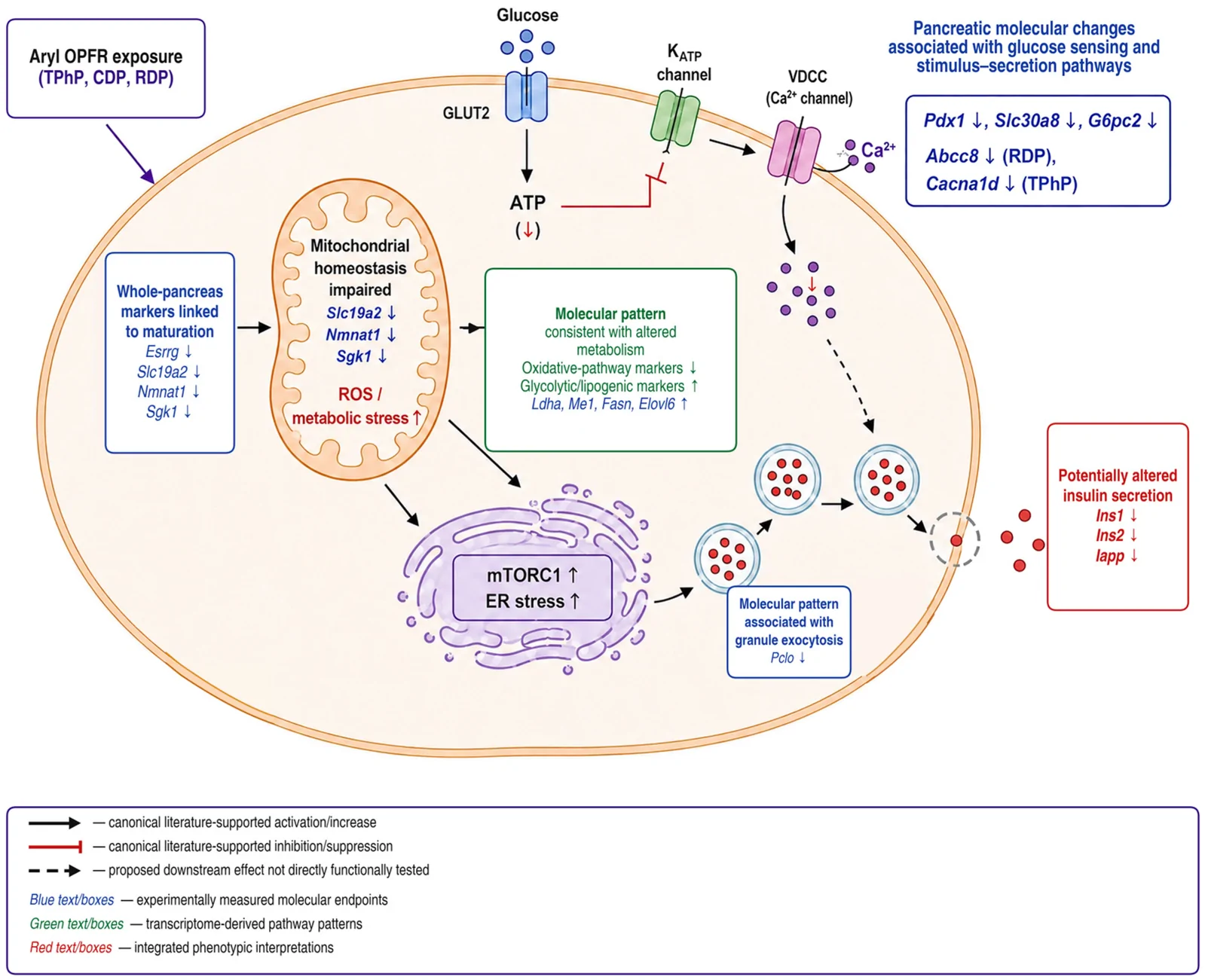
Evidence-integrated model linking whole-pancreas molecular changes after TPhP, CDP, and RDP exposure to pancreatic metabolic and secretion-associated pathways. Blue labels/boxes denote experimentally measured molecular endpoints; green labels/boxes denote transcriptome-derived pathway patterns; red labels/boxes denote integrated phenotypic interpretations. Solid arrows show canonical literature-supported relationships, whereas dashed arrows show proposed downstream effects not directly tested. T-ended lines denote inhibition or suppression; upward and downward arrows beside labels indicate increases and decreases, respectively. Blue, purple, and red circles represent glucose, calcium ions, and insulin, respectively; outlined vesicles represent secretory granules. Cell and organelle icons are schematic, and boxes group endpoints or interpretations. The ESRRG–LDHA and secretion-associated links are hypothesis-generating. Neither a causal relationship between ESRRG and LDHA nor a β-cell-specific mechanism or functional secretion defect was established.

**Table 1:** Exposure groups and dosing regimen.

Group	Compound	Time-Weighted Average Dose	Dose per Administration (Every 3 Days)
Control	Vehicle	0	Corn oil only
TPhP-L	TPhP	20 ng/kg bw/day	60 ng/kg bw
TPhP-H	TPhP	20 μg/kg bw/day	60 μg/kg bw
CDP-L	CDP	20 ng/kg bw/day	60 ng/kg bw
CDP-H	CDP	20 μg/kg bw/day	60 μg/kg bw
RDP-L	RDP	20 ng/kg bw/day	60 ng/kg bw
RDP-H	RDP	20 μg/kg bw/day	60 μg/kg bw

## Data Availability

The data supporting the findings of this study are available within the article and its Supplementary Materials. The raw data, including the transcriptomic datasets, are available from the corresponding authors upon reasonable request.

## Supplementary Materials

The following supporting information can be downloaded at https://www.mdpi.com/article/10.3390/toxics14100841/s1. Table S1: Physicochemical properties of TPhP, CDP, and RDP; Table S2: Commercial assay kits used for serum, plasma, liver, and pancreas measurements; Table S3: Primary antibodies used for Western blotting; Table S4: Comparison of reported glucose-metabolic, tissue, and molecular endpoints after TPhP, CDP, and RDP exposure; Text S1: Commercial Assay Kits and Biochemical Measurements; Text S2: Acute Insulin Stimulation and Immunoblotting; Text S3. Gene Selection and Data Preprocessing for Pancreatic Marker-Gene PCA; Figure S1: Original overall-transcriptome PCA of the pancreatic RNA-sequencing dataset; Figure S2: Plasma AST and ALT levels in mice exposed to TPhP, CDP, and RDP; Figure S3: H&E-stained liver sections from wild-type mice exposed to TPhP, CDP, and RDP at 100× magnification; Figure S4: High-magnification H&E-stained liver sections from wild-type mice exposed to TPhP, CDP, and RDP; Figure S5: Low-magnification Oil Red O-stained liver sections from mice exposed to TPhP, CDP, and RDP; Figure S6: High-magnification Oil Red O-stained liver sections from mice exposed to TPhP, CDP, and RDP; Figure S7: Blinded semiquantitative analysis of the Oil Red O-positive area in liver after TPhP, CDP, and RDP exposure; Figure S8: High-magnification H&E-stained pancreatic islet fields from wild-type mice exposed to TPhP, CDP, and RDP; Figure S9: Exploratory GSEA enrichment plots of representative metabolic pathways in liver after higher-level TPhP, CDP, or RDP exposure; Figure S10: Effects of higher-level TPhP, CDP, and RDP exposure on hepatic mRNA expression; Figure S11: Exploratory whole-pancreas GSEA enrichment plots; Figure S12: Effects of higher-level TPhP, CDP, and RDP exposure on whole-pancreas mRNA expression.
