# Supplementary material for: CDP and RDP as Alternatives to TPhP: Comparative Effects on Glucose Homeostasis and Hepatic and Whole-Pancreas Molecular Endpoints in Mice
Source: Toxics. 2026 Sep 22;14(10):841. doi: 10.3390/toxics14100841 (PMC13709551; doi:10.3390/toxics14100841)
Supplement: Supplementary file 1 [file toxics-14-00841-s001.zip › toxics-4510943-supplementary.pdf]

**Supporting information for**

# CDP and RDP as Alternatives to TPhP: Comparative Effects on Glucose Homeostasis and Hepatic and Whole-Pancreas Molecular Endpoints in Mice

**Jiajun Chang [1,2], Zhuozhuo Wang [1,2], Yao Xiao [1,2], Yifan Liu [3], Yuan Meng [4], Qinghui Huang [1,2], Zhiliang Zhu [1,2], Daqiang Yin [1,2], Xiaojuan Xu [3,*] and Yanling Qiu [1,2,*]**

[1] Key Laboratory of Yangtze River Water Environment, College of Environmental Science and Engineering, Tongji University, 1239 Siping Road, Shanghai 200092, China; 2210091@tongji.edu.cn (J.C.);
2210526@tongji.edu.cn (Z.W.); 2111065@tongji.edu.cn (Y.X.); qhhuang@tongji.edu.cn (Q.H.); zzl@tongji.edu.cn (Z.Z.); yindq@tongji.edu.cn (D.Y.)

[2] Shanghai Institute of Pollution Control and Ecological Security, College of Environmental Science and Engineering, Tongji University, 1239 Siping Road, Shanghai 200092, China;

[3] Department of Pathology and Pathophysiology, Teaching Laboratory Center, School of Medicine, Tongji University, Shanghai 200331, China;
liuyifan170909@163.com

[4] Department of Criminal Science and Technology, Shandong Police College, Jinan 250200, China;
yuanmeng0419@163.com

* Correspondence: xiaojuanxu@tongji.edu.cn (X.X.); ylqiu@tongji.edu.cn (Y.Q.);
Tel.: +86-13795329386 (X.X.); Tel.: +86-13916924371 (Y.Q.)

**Table S1. Physicochemical properties of TPhP, CDP, and RDP.**

| Compound | Abbreviation | Molecular weight (g/mol) | log $K_{ow}$ | Predicted BCF |
|---|---|---|---|---|
| Triphenyl phosphate | TPhP | 326.28 | 4.59 | 113 |
| Cresyl diphenyl phosphate | CDP | 340.32 | 5.25 | $1.71 \times 10^3$ |
| Resorcinol bis(diphenyl phosphate) | RDP | 574.45 | 7.41 | $2.05 \times 10^4$ |

Note: BCF, bioconcentration factor; Kow, octanol–water partition coefficient. Predicted fish BCF values were generated using the regression-based method in BCFBAF v3.01 within US EPA EPI Suite v4.11. These screening-level estimates were not measured in the present mice and should not be interpreted as internal-exposure data.

**Table S2. Commercial assay kits used for serum, plasma, liver, and pancreas measurements.**

| Endpoint | Sample | Assay format | Manufacturer | Catalog no. |
|---|---|---|---|---|
| Glycogen | Liver homogenate | Mouse ELISA, 96-well | Shanghai Enzyme-linked Biotechnology Co., Ltd., Shanghai, China | ml037785 |
| Insulin receptor (IR) | Liver homogenate | Mouse sandwich ELISA, 96-well | Shanghai Enzyme-linked Biotechnology Co., Ltd., Shanghai, China | ml058257 |
| Insulin receptor substrate 1 (IRS-1) | Liver homogenate | Mouse sandwich ELISA, 96-well | Shanghai Enzyme-linked Biotechnology Co., Ltd., Shanghai, China | ml058448 |
| GLUT1 | Liver and pancreas homogenates | Mouse sandwich ELISA, 96-well | Shanghai Enzyme-linked Biotechnology Co., Ltd., Shanghai, China | ml037552 |
| GLUT4 | Liver homogenate | Mouse sandwich ELISA, 96-well | Shanghai Enzyme-linked Biotechnology Co., Ltd., Shanghai, China | ml037551 |
| M-AChR | Pancreas homogenate | Mouse sandwich ELISA, 96-well | Shanghai Enzyme-linked Biotechnology Co., Ltd., Shanghai, China | ml037816 |
| Insulin | Serum | Mouse sandwich ELISA, 96-well | Shanghai Enzyme-linked Biotechnology Co., Ltd., Shanghai, China | ml001983 |
| ALT | Plasma | Mouse sandwich ELISA, 96-well | Shanghai Enzyme-linked Biotechnology Co., Ltd., Shanghai, China | ml063179 |
| AST | Plasma | Mouse sandwich ELISA, 96-well | Shanghai Enzyme-linked Biotechnology Co., Ltd., Shanghai, China | ml058659 |
| ATP | Liver and pancreas homogenates | Luciferase-based chemiluminescence | Beyotime Biotechnology, Shanghai, China | S0027 |

**Table S3. Primary antibodies used for Western blotting.**

| Target | Manufacturer | Catalog no. | Working dilution |
|---|---|---|---|
| Total AKT | ProMabio | P17171 | 1:5000 |
| Phospho-AKT (Ser473) | Proteintech | 28731-1-AP | 1:1000 |
| Phospho-AKT (Thr308) | Proteintech | 29163-1-AP | 1:1000 |
| GYS2 | Proteintech | 22371-1-AP | 1:2000 |
| GLUT4 | Abcam | ab216661 | 1:1000 |
| ESRRG | Invitrogen | PA5-27755 | 1:1000 |
| LDHA | Proteintech | 19987-1-AP | 1:2000 |
| β-Actin (HRP-conjugated) | Proteintech | HRP-60008 | 1:5000 |

**Table S4. Comparison of reported glucose-metabolic, tissue, and molecular endpoints after TPhP, CDP, and RDP exposure.**

**A. Systemic and biochemical endpoints**

| Endpoint | TPhP | CDP | RDP | Source |
|---|---|---|---|---|
| OGTT AUC | L ↑*; H NS | L NS; H ↑* | L NS; H ↑* | Fig. 1d |
| ITT AUC | L/H NS | L ↑*; H NS | L/H ↑* | Fig. 1f |
| Basal serum insulin | L NS; H ↓* | L/H ↓* | L NS; H ↓* | Fig. 1e |
| Serum insulin at 30 min | H: lower mean | L/H: lower means | L/H: lower means | Fig. 1e |
| Liver glycogen | L NS; H ↓* | L NS; H ↓* | L NS; H ↓* | Fig. 2a |
| Liver IR | L NS; H ↓* | L/H ↓* | L NS; H ↓* | Fig. 2b |
| Liver GLUT1 (ELISA) | L NS; H ↓* | L NS; H ↓* | L ↑*; H ↓* | Fig. 2c |
| Liver IRS-1 | L NS; H ↓* | L NS; H ↓* | L NS; H ↓* | Fig. 2d |
| Liver ATP | L NS; H ↓* | L/H ↓* | L NS; H ↓* | Fig. 2e |
| Liver GLUT4 (ELISA) | L NS; H ↓* | L NS; H ↓* | L NS; H ↓* | Fig. 2f |
| Pancreas GLUT1 | L ↑*; H ↓* | L/H NS | L NS; H ↓* | Fig. 3a |
| Pancreas M-AChR | L NS; H ↓* | L/H NS | L NS; H ↓* | Fig. 3b |
| Pancreas ATP | L NS; H ↓* | L NS; H ↓* | L/H NS; lower H mean | Fig. 3c |
| Plasma AST | L/H: higher means; no significance marked | L/H ↑* | L/H ↑* | Fig. S1a |
| Plasma ALT | L NS; H ↑* | L NS; H ↑* | L/H ↑* | Fig. S1b |
| Liver Oil Red O-positive area | L/H ↑* | L/H ↑* | L/H ↑*; largest displayed L mean | Fig. S6 |

L and H denote time-weighted average doses of 20 ng/kg body weight/day and 20 µg/kg body weight/day, delivered as 60 ng/kg or 60 µg/kg body weight by gavage every 3 days for 70 days. ↑ and ↓ indicate the reported direction relative to the relevant Control. An asterisk denotes a statistically significant comparison identified in the source figure; it does not encode a common effect size or exact P value. NS indicates no statistically significant difference identified in the source figure and is not evidence of equivalence. Statements about means are descriptive. For post-insulin phospho-AKT, the comparator is Control+INS. OGTT, ITT, and serum insulin used n = 6 per group; biochemical, protein, histological, and qRT-PCR endpoints used n = 3. Pancreatic RNA sequencing used Control n = 3 and each exposed group n = 2. Gene-expression and pathway entries in part C refer to the higher dose only. DEG counts and pathway patterns are exploratory; they do not measure toxicity severity. This table summarizes existing results and introduces no additional statistical comparisons between compounds.

**B. Protein and histological endpoints**

| Endpoint | TPhP | CDP | RDP | Source |
|---|---|---|---|---|
| Liver GYS2 protein | L/H NS; lower means | L/H ↓* | L/H ↓* | Fig. 6d |
| Liver GLUT4 protein | L/H NS | L/H NS | L/H NS | Fig. 6e |
| Pancreas ESRRG protein | L/H ↓* | L/H ↓* | L/H ↓* | Fig. 6f |
| Pancreas LDHA protein | L/H ↑* | L/H ↑* | L/H ↑* | Fig. 6g |
| Liver total AKT | L/H NS | L/H NS | L/H NS | Fig. 6h |
| Liver post-insulin p-AKT (Thr308) | L/H ↓* | L/H ↓* | L/H ↓* | Fig. 6i |
| Liver post-insulin p-AKT (Ser473) | L ↓*; H NS | L/H ↓* | L/H ↓* | Fig. 6j |
| Liver H&E morphology | Descriptive fields; no quantitative severity score | Descriptive fields; no quantitative severity score | Descriptive fields; no quantitative severity score | Figs. S2, S3 |
| Pancreas H&E morphology | No widespread destructive changes reported | No widespread destructive changes reported | No widespread destructive changes reported | Fig. S7 |

Abbreviations, sample sizes, statistical notation, and interpretive limits are defined in the note to Table S4, part A.

**C. Higher-level gene-expression and pathway endpoints**

| Endpoint | TPhP | CDP | RDP | Source |
|---|---|---|---|---|
| Liver *Socs3*, *Nos2*, *Srebf1*, *Pck1*, *Nfkbia* | ↑* | ↑* | ↑* | Fig. S9a,c–f |
| Liver *Ppp1r3b* | ↓* | NS; lower mean | ↓* | Fig. S9b |
| Liver *Tbc1d4* (AS160) | NS; lower mean | NS; lower mean | ↓* | Fig. S9g |
| Liver *Gys2* | ↓* | ↓* | ↓* | Fig. S9h |
| Liver *Slc2a2* | ↓* | NS; lower mean | ↓* | Fig. S9i |
| Pancreas *Esrrg*, *Pclo*, *Sgk1*, *Slc19a2* | ↓* | ↓* | ↓* | Fig. S11a–d |
| Pancreas *Nmnat1* | NS; lower mean | ↓* | ↓* | Fig. S11e |
| Pancreas *Ldha*, *Me1*, *Fasn*, *Elovl6* | ↑* | ↑* | ↑* | Fig. S11f–i |
| Pancreas screened DEGs | 498 (440 up; 58 down) | 250 (172 up; 78 down) | 352 (290 up; 62 down) | Fig. 5a–c |
| Selected liver GSEA patterns | Negative starch/sucrose, fatty-acid degradation and PPAR patterns | Altered pentose-phosphate and fatty-acid-degradation patterns | Negative peroxisome and fatty-acid-degradation patterns | Fig. S8 |
| Selected pancreas GSEA patterns | Protein secretion and E2F targets | Insulin secretion and mTORC1 programs | Glycolysis and oxidative phosphorylation | Fig. S10 |

Abbreviations, sample sizes, statistical notation, and interpretive limits are defined in the note to Table S4, part A.

**Text S1. Commercial Assay Kits and Biochemical Measurements**

Serum and EDTA-anticoagulated plasma were separated, aliquoted, and stored at −80 °C. Frozen liver or pancreas was homogenized in pre-cooled PBS or the assay-specific lysis buffer at approximately 1:9 (w/v) and centrifuged at 4 °C, and the supernatant was retained. Total protein was measured by the BCA method. ELISA kits were used in accordance with the manufacturer-specific instructions. Standards, samples, and blanks were loaded into designated wells, followed by incubation with the supplied detection reagents, plate washing, tetramethylbenzidine color development, and addition of stop solution. Absorbance was read at 450 nm, and sample concentrations were calculated from the corresponding standard curves after applying the required dilution factors. Tissue endpoints were normalized to total protein where applicable.

For ATP determination, tissue was homogenized in pre-cooled ATP lysis buffer and centrifuged at approximately 12,000×g for 5 min at 4 °C. ATP working solution (100 μL) was dispensed into each well of a white 96-well plate and equilibrated for 3–5 min. ATP standard or tissue supernatant (20 μL) was then added, luminescence was measured immediately, and ATP concentrations were calculated from the standard curve and normalized to total protein.

**Text S2. Acute Insulin Stimulation and Immunoblotting**

For analysis of hepatic AKT phosphorylation after insulin, mice received an acute intraperitoneal injection of insulin at 0.25 U/kg body weight, and liver samples were collected 30 min later. The design included unstimulated Control and insulin-stimulated Control+INS groups, whereas each OPFR-exposed group was sampled only after insulin. Comparisons with Control+INS describe insulin signaling-related changes after insulin administration. Without basal exposed groups, they cannot distinguish altered basal phosphorylation from an attenuated insulin response or directly establish hepatic insulin resistance. Each independent animal was treated as one biological replicate; technical measurements were averaged within each animal.

**Text S3. Gene Selection and Data Preprocessing for Pancreatic Marker-Gene PCA**

Pancreatic marker-gene PCA used a manually curated panel spanning acinar, ductal, endocrine, endothelial, stellate/fibroblast, and immune-cell-associated compartments. Genes were chosen for their established cell-identity or compartment-associated functions and their availability in the expression matrix, not for differential-expression significance, fold change, or treatment-group separation. The panel comprised 118 available genes, grouped below. TPM values were transformed as log2(TPM + 1), and each gene was centered and scaled to unit variance across the nine samples before PCA. All nine pancreatic samples were retained. Restricting features to this panel focused the visualization on pancreatic cell-associated expression but did not remove contaminating cells, estimate cell proportions, or make the analysis β-cell-specific.

Acinar-associated genes (26): *Prss1*, *Prss2*, *Pnlip*, *Pnliprp1*, *Pnliprp2*, *Cpa1*, *Cpa2*, *Cpb1*, *Amy2a1*, *Amy2a2*, *Cela1*, *Cela2a*, *Cela3a*, *Cela3b*, *Ctrb1*, *Try4*, *Try5*, *Bhlha15*, *Rbpjl*, *Ptf1a*, *Nr5a2*, *Sycn*, *Pla2g1b*, *Ctrl*, *Cel*, *Spink1*.

Ductal-associated genes (16): *Krt8*, *Krt18*, *Krt19*, *Krt7*, *Sox9*, *Muc1*, *Spp1*, *Krt17*, *Cftr*, *Klf5*, *Epcam*, *Hnf1b*, *Onecut1*, *Mmp7*, *Prom1*, *Clu*.

Endocrine-associated genes (24): *Ins1*, *Ins2*, *Gcg*, *Sst*, *Ppy*, *Iapp*, *Pcsk1*, *Pcsk2*, *Isl1*, *Nkx6-1*, *Pdx1*, *Mafa*, *Mafb*, *Arx*, *Hhex*, *Chga*, *Chgb*, *Scg2*, *Scg3*, *Scg5*, *Abcc8*, *Kcnj11*, *G6pc2*, *Ucn3*.

Endothelial-associated genes (14): *Kdr*, *Esam*, *Emcn*, *Pecam1*, *Cdh5*, *Egfl7*, *Klf2*, *Eng*, *Rbp7*, *Esm1*, *Plvap*, *Tek*, *Vwf*, *Aqp1*.

Stellate/fibroblast-associated genes (20): *Col1a1*, *Col1a2*, *Col3a1*, *Col5a1*, *Col5a2*, *Dcn*, *Lum*, *Col6a1*, *Col6a2*, *Col6a3*, *Pdgfra*, *Pdgfrb*, *Rgs5*, *Cspg4*, *Acta2*, *Tagln*, *Col14a1*, *Pi16*, *C7*, *C3*.

Immune-cell-associated genes (18): *Ptprc*, *Lyz2*, *Cd68*, *Adgre1*, *Tyrobp*, *Lst1*, *C1qa*, *C1qb*, *C1qc*, *Cd3d*, *Cd3e*, *Cd79a*, *Ms4a1*, *Nkg7*, *Ccl5*, *Mrc1*, *Fcgr3*, *Itgam*.

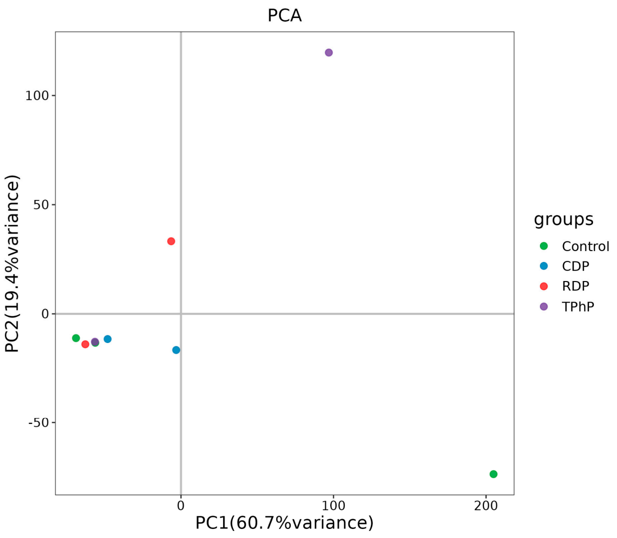


Figure S1. Original overall-transcriptome PCA of the pancreatic RNA-sequencing dataset. PC1 and PC2 explain 60.7% and 19.4% of the variance, respectively, as displayed in the original analysis output. Each point represents one biological sample. The original plot is reproduced for comparison with the 118-marker PCA in Figure 5d; sample positions, labels, and variance percentages have been retained. The broad within-group dispersion and overlap indicate expression heterogeneity, and this descriptive plot does not establish robust treatment separation or a β-cell-specific response.

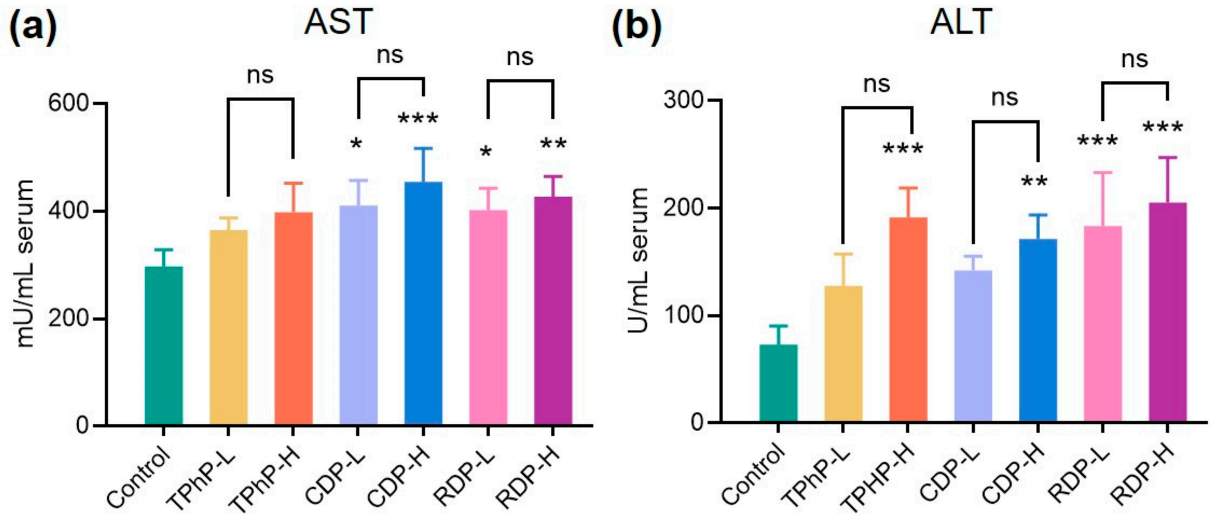


Figure S2. Plasma AST and ALT levels in mice exposed to TPhP, CDP, and RDP. (a) AST and (b) ALT. Data are mean ± SD (n = 3 independent animals per group; technical measurements were averaged within each animal). * $P < 0.05$, ** $P < 0.01$, *** $P < 0.001$, and **** $P < 0.0001$ versus Control; ns, not significant.

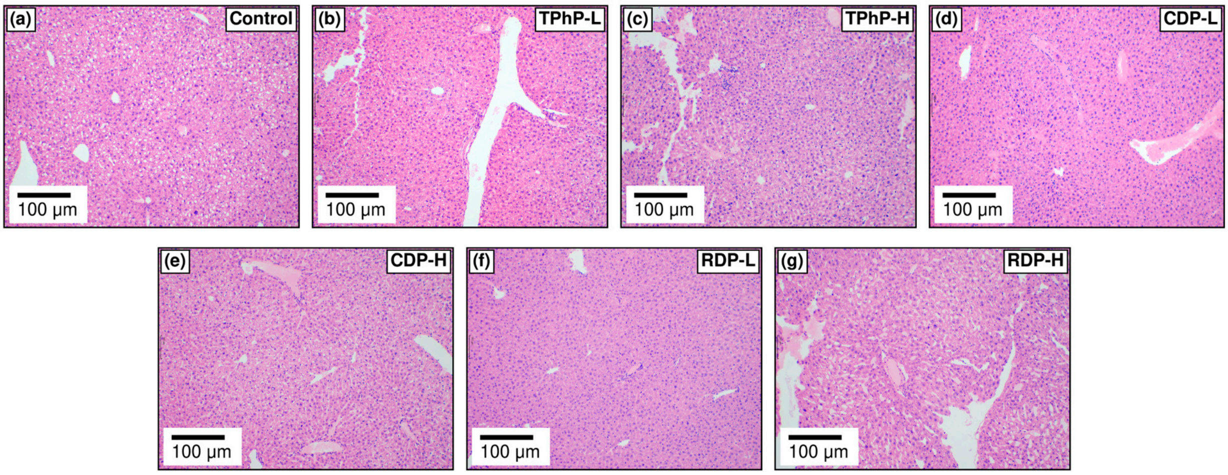


Figure S3. H&E-stained liver sections from wild-type mice exposed to TPhP, CDP, and RDP at 100× magnification. Scale bar = 100 µm. Images are representative descriptive fields from n = 3 independent animals per group.

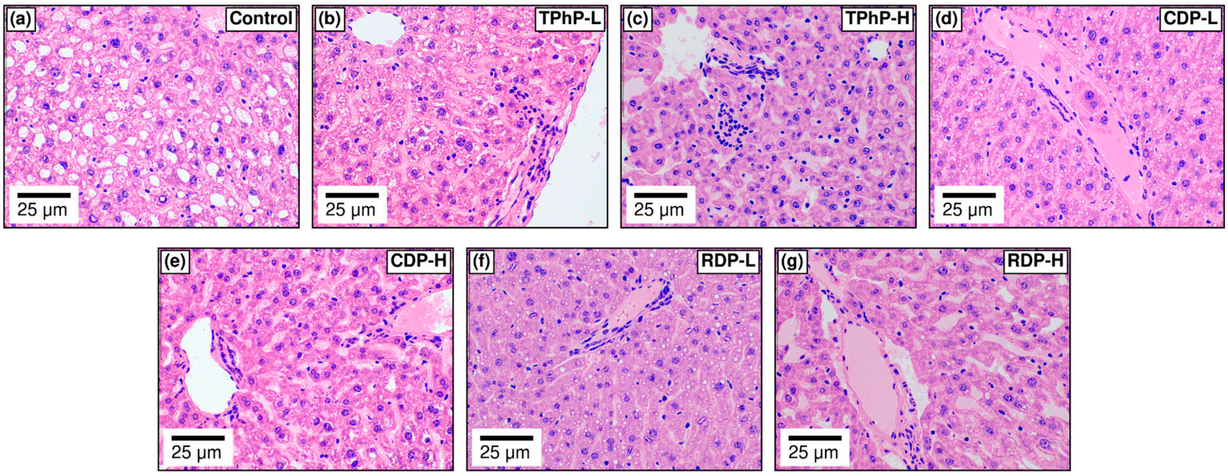


Figure S4. High-magnification H&E-stained liver sections from wild-type mice exposed to TPhP, CDP, and RDP (400×; scale bar = 25 µm). Images are representative descriptive fields from n = 3 independent animals per group.

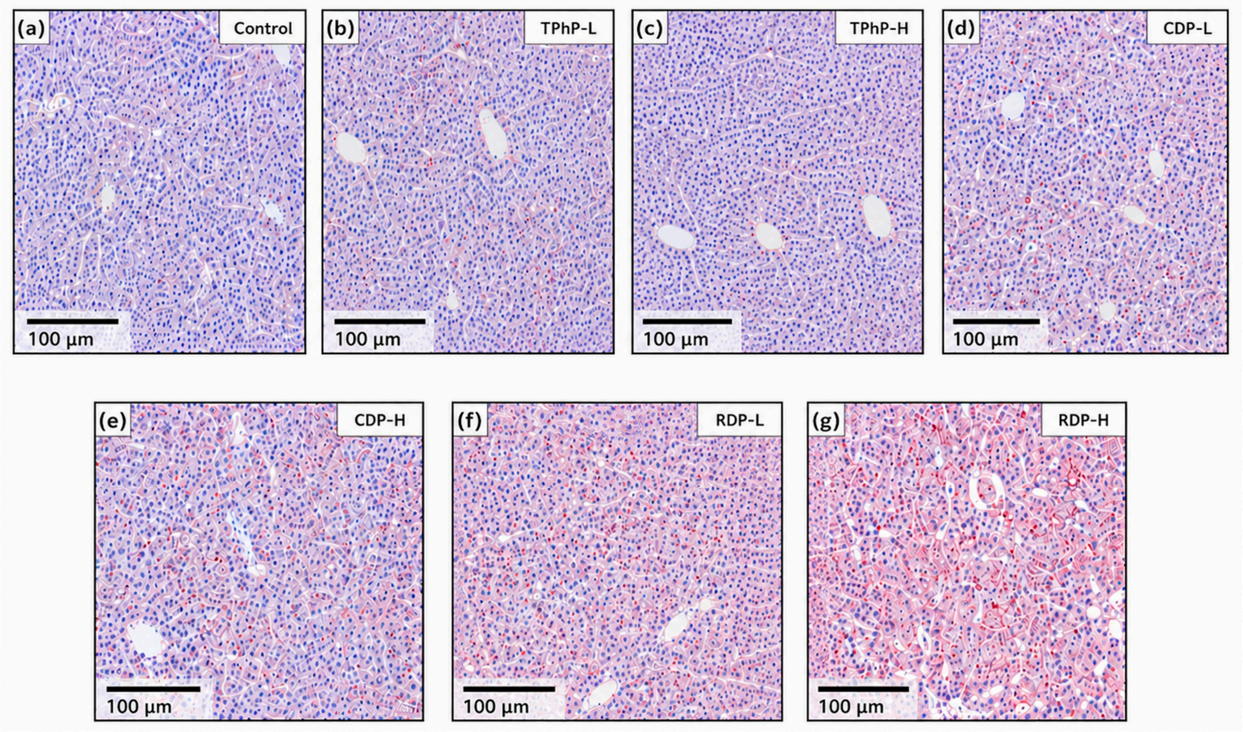


Figure S5. Low-magnification Oil Red O-stained liver sections from mice exposed to TPhP, CDP, and RDP (100×; scale bar = 100 µm). Images are representative descriptive fields from n = 3 independent animals per group.

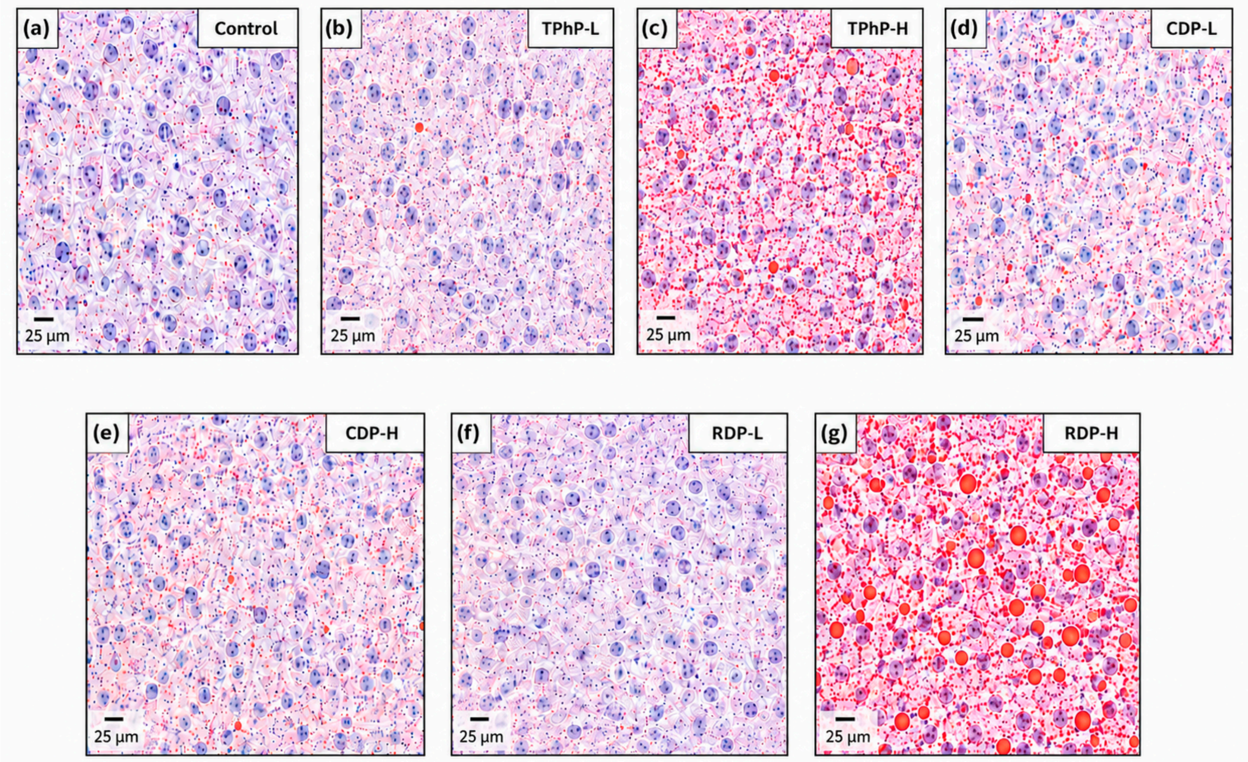


Figure S6. High-magnification Oil Red O-stained liver sections from mice exposed to TPhP, CDP, and RDP (400×; scale bar = 25 µm). Images are representative descriptive fields from n = 3 independent animals per group and illustrate cellular lipid-droplet distribution.

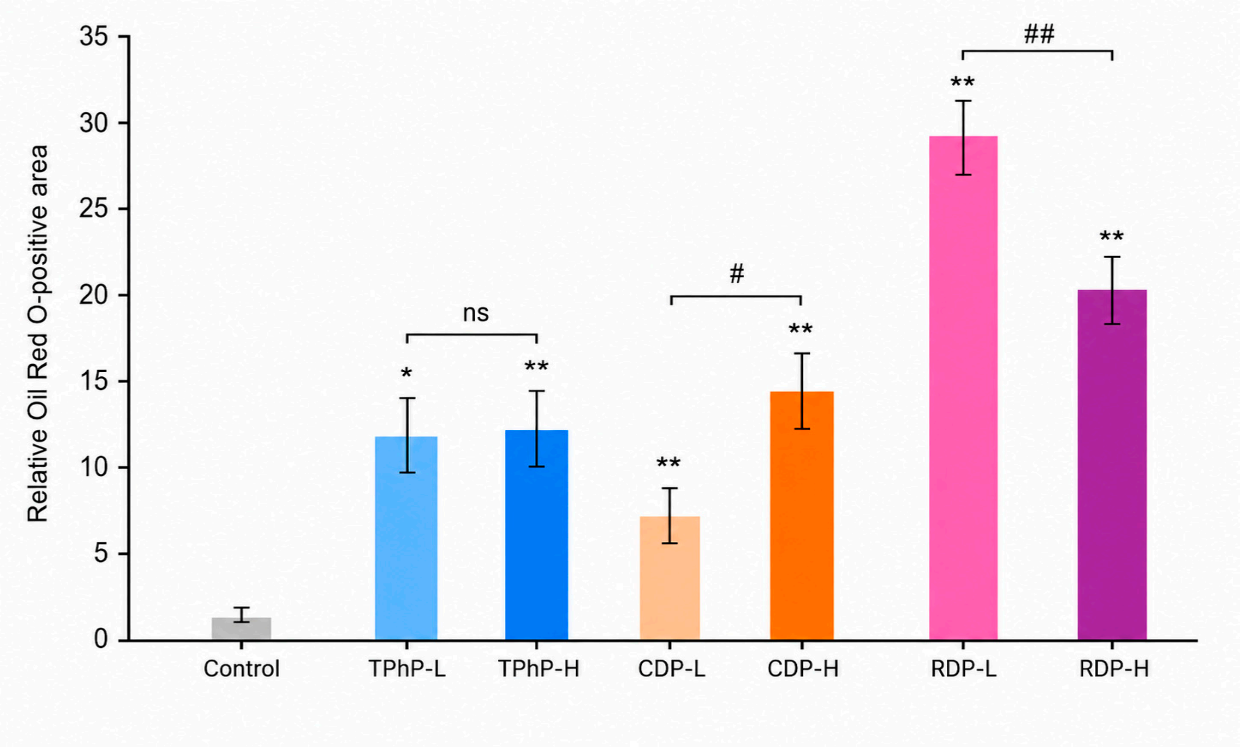


Figure S7. Blinded semiquantitative analysis of the Oil Red O-positive area in liver after TPhP, CDP, and RDP exposure. Group codes were masked during ImageJ analysis. Data are mean ± SD (n = 3 independent animals per group; multiple fields were averaged within each animal and were not treated as independent n values). * P < 0.05 and ** P < 0.01 versus Control; # P < 0.05 and ## P < 0.01 between corresponding lower- and higher-level groups; ns, not significant.

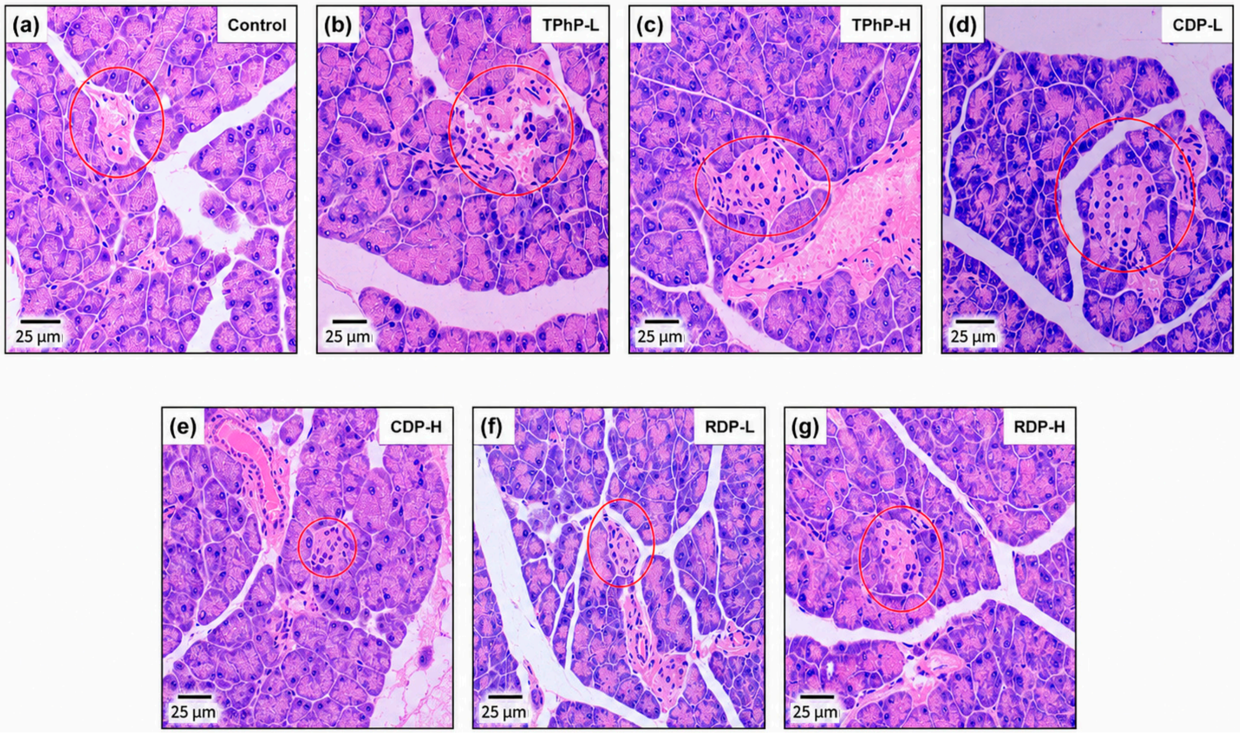


Figure S8. High-magnification H&E-stained pancreatic islet fields from wild-type mice exposed to TPhP, CDP, and RDP (400×; scale bar = 25 μm). Red circles indicate pancreatic islets. Images are presented descriptively; no field-level cell-count inference is made.

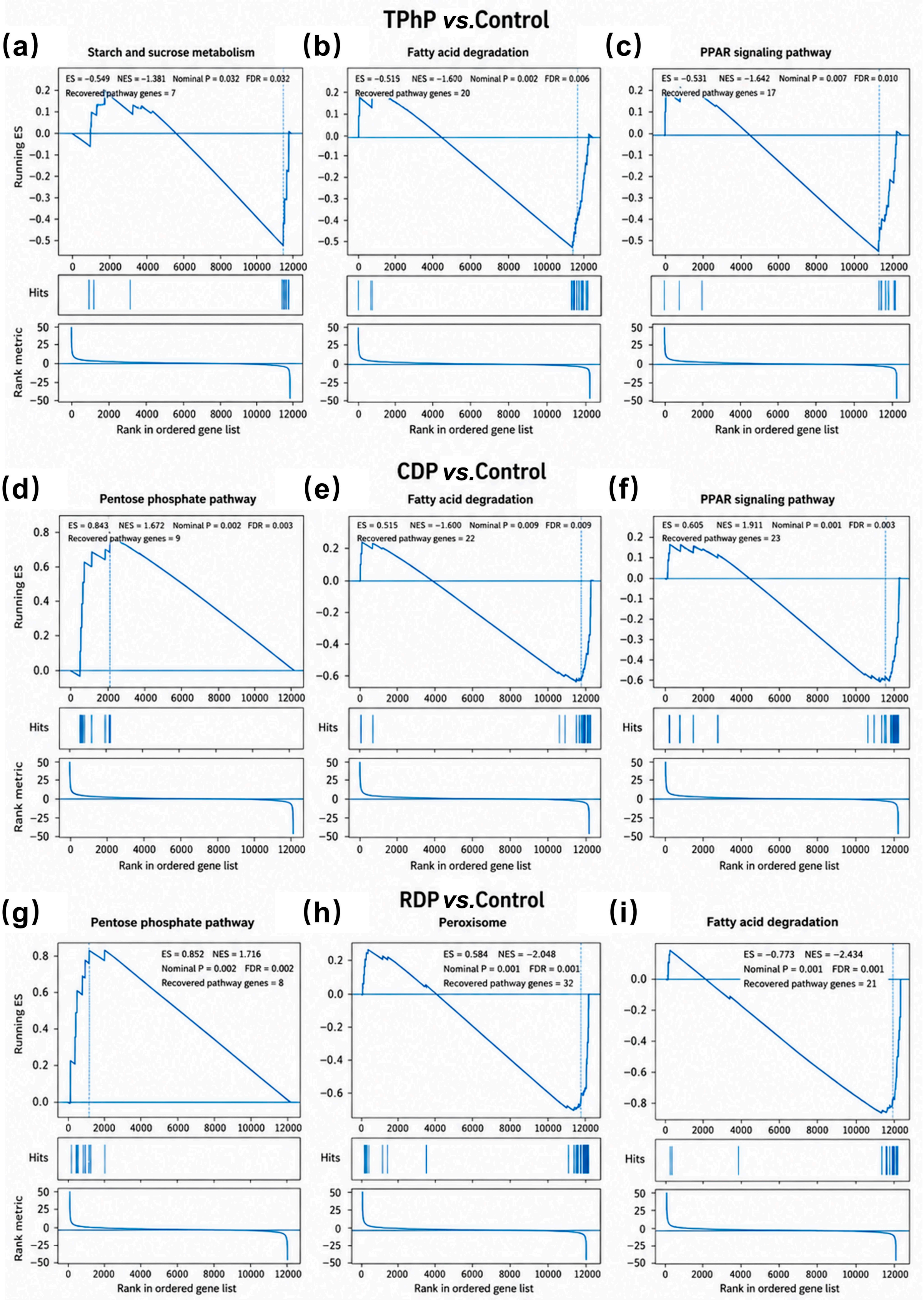


Figure S9. Exploratory GSEA enrichment plots of representative metabolic pathways in liver after higher-level TPhP, CDP, or RDP exposure. (a–c) TPhP; (d–f) CDP; and (g–i) RDP. Analysis used all genes ranked by the DESeq2 Wald statistic and 1000 gene-set permutations rather than phenotype-label permutations. ES, NES, nominal P value, and FDR are shown. With n = 3 biological replicates per group, these plots are hypothesis-generating.

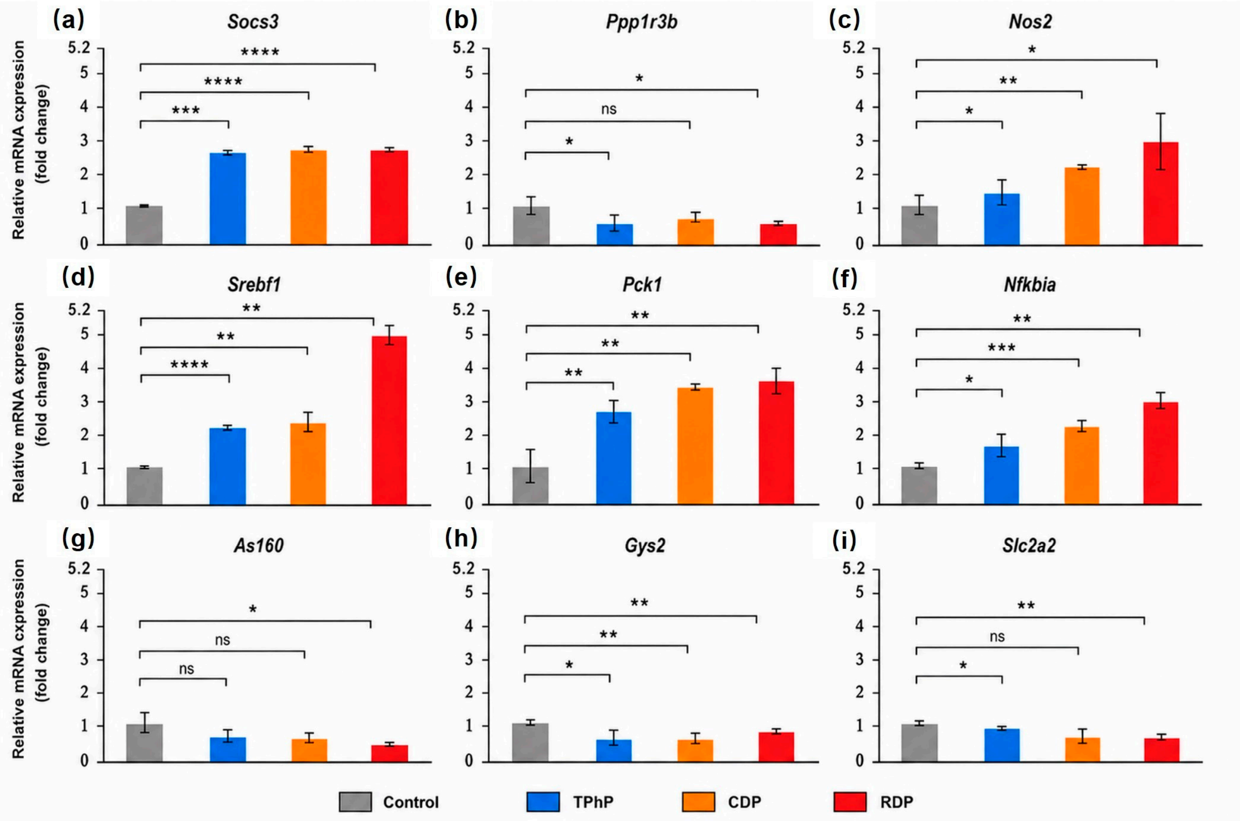


Figure S10. Effects of higher-level TPhP, CDP, and RDP exposure on hepatic mRNA expression. (a–i) *Socs3*, *Ppp1r3b*, *Nos2*, *Srebf1*, *Pck1*, *Nfkbia*, *Tbc1d4* (AS160), *Gys2*, and *Slc2a2*. Data are mean ± SD (n = 3 independent animals per group). Three technical wells were averaged within each animal and were not counted as independent replicates. * P < 0.05, ** P < 0.01, *** P < 0.001, and **** P < 0.0001 versus Control; ns, not significant.

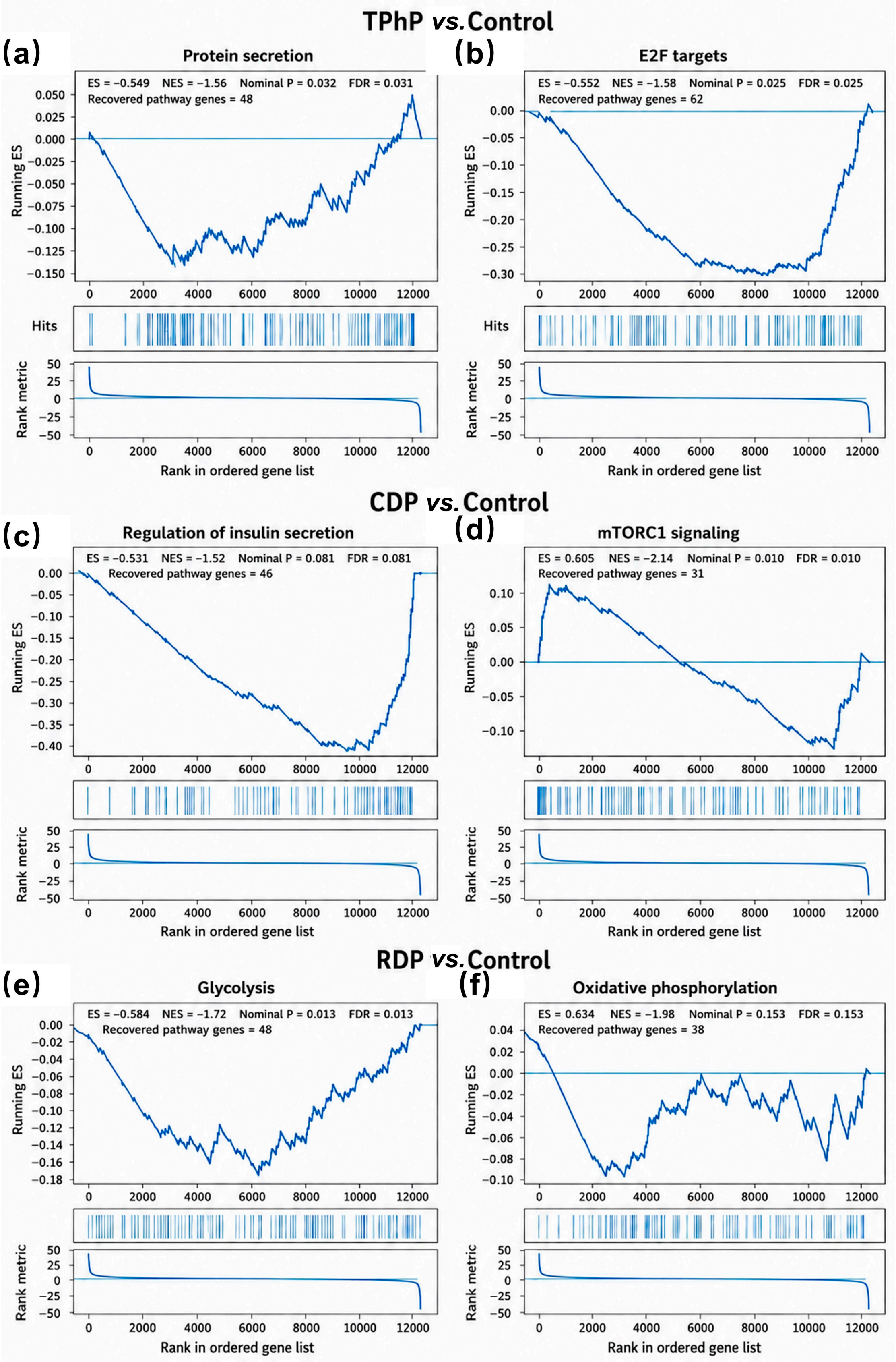


Figure S11. Exploratory whole-pancreas GSEA enrichment plots. (a,b) Protein secretion and E2F targets after TPhP; (c,d) regulation of insulin secretion and mTORC1 signaling after CDP; and (e,f) glycolysis and oxidative phosphorylation after RDP. Analysis used all genes ranked by the DESeq2 Wald statistic and 1000 gene-set permutations rather than phenotype-label permutations. ES, NES, nominal P value, and FDR are shown. With three Control samples, two biological replicates per exposed group, and bulk-tissue sampling, the results are hypothesis-generating and not β-cell-specific.

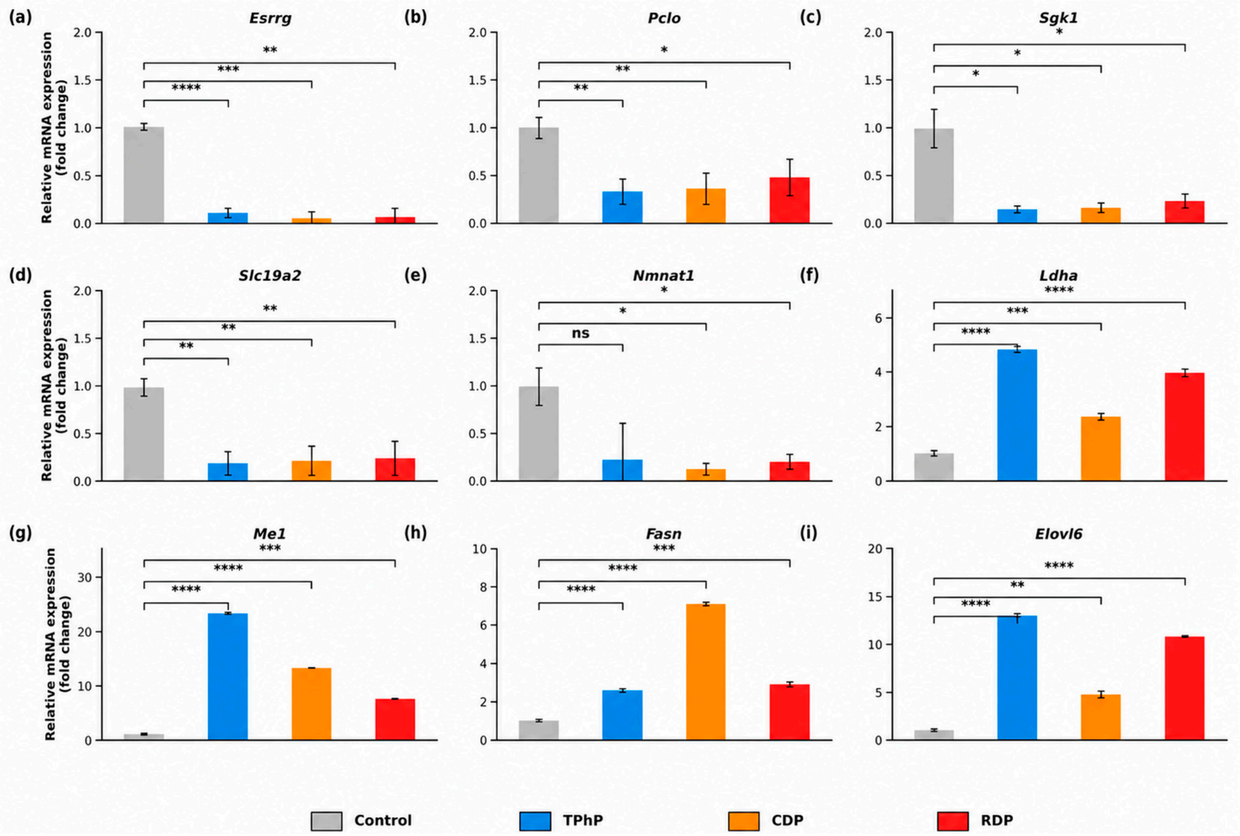


Figure S12. Effects of higher-level TPhP, CDP, and RDP exposure on whole-pancreas mRNA expression. (a) *Esrrg*; (b) *Pclo*; (c) *Sgk1*; (d) *Slc19a2*; (e) *Nmnat1*; (f) *Ldha*; (g) *Me1*; (h) *Fasn*; and (i) *Elovl6*. Data are mean ± SD (n = 3 independent animals per group). Three technical wells were averaged within each animal and were not counted as independent replicates. * $P < 0.05$, ** $P < 0.01$, *** $P < 0.001$, and **** $P < 0.0001$ versus Control; ns, not significant. Bulk-tissue changes are not β-cell-specific.
